# Microalgal TAG production strategies: why batch beats repeated-batch

**DOI:** 10.1186/s13068-016-0475-4

**Published:** 2016-03-16

**Authors:** Giulia Benvenuti, Packo P. Lamers, Guido Breuer, Rouke Bosma, Ana Cerar, René H. Wijffels, Maria J. Barbosa

**Affiliations:** Bioprocess Engineering, AlgaePARC, Wageningen University, P.O. Box 16, 6700 AA Wageningen, The Netherlands; Microbiology, Food Science and Technology, Biotechnical Faculty, University of Ljubljana, Večna pot 111, 1000 Ljubljana, Slovenia; Biosciences and Aquaculture, Nordland University, 8049 Bodø, Norway

**Keywords:** Microalgae, TAG production, Batch, Repeated-batch, Recovery, Mechanistic model

## Abstract

**Background:**

For a commercially feasible microalgal triglyceride (TAG) production, high TAG productivities are required. The operational strategy affects TAG productivity but a systematic comparison between different strategies is lacking. For this, physiological responses of *Nannochloropsis* sp. to nitrogen (N) starvation and N-rich medium replenishment were studied in lab-scale batch and repeated-batch (part of the culture is periodically harvested and N-rich medium is re-supplied) cultivations under continuous light, and condensed into a mechanistic model.

**Results:**

The model, which successfully described both strategies, was used to identify potential improvements for both batch and repeated-batch and compare the two strategies on optimized TAG yields on light (amount of TAGs produced per mol of supplied PAR photons). TAG yields on light, for batch, from 0.12 (base case at high light) to 0.49 g mol_ph_^−1^ (at low light and with improved strain) and, for repeated-batch, from 0.07 (base case at high light) to 0.39 g mol_ph_^−1^ (at low light with improved strain and optimized repeated-batch settings). The base case yields are in line with the yields observed in current state-of-the-art outdoor TAG production.

**Conclusions:**

For continuous light, an optimized batch process will always result in higher TAG yield on light compared to an optimized repeated-batch process. This is mainly because repeated-batch cycles start with N-starved cells. Their reduced photosynthetic capacity leads to inefficient light use during the regrowth phase which results in lower overall TAG yields compared to a batch process.

**Electronic supplementary material:**

The online version of this article (doi:10.1186/s13068-016-0475-4) contains supplementary material, which is available to authorized users.

## Background

Triglycerides (TAGs) are a class of non-polar lipids that are regarded as a sustainable feedstock for the chemical, food, and biofuel industries [1–3]. In microalgae, TAGs are accumulated under unfavorable growth conditions (e.g., high light intensities and/or nitrogen limitation/starvation), leading to a reduction in TAG productivity over time [4–6]. TAG production is often carried out in a two-phase process in which biomass is first produced under nitrogen (N) replete conditions and then TAGs are accumulated under N-depleted conditions in batch-operated cultivations [7, 8]. In our previous study (Benvenuti et al. manuscript submitted), lab-scale repeated-batch cultivations (during which part of the culture is periodically harvested and N-rich medium is re-supplied) were investigated leading to similar TAG productivities compared to batch cultivations. Nevertheless, a full optimization of repeated-batch TAG production is still lacking whereas a systematic process comparison is needed. For this, understanding of cell recovery mechanisms upon N-rich medium resupply is necessary as such recovery may greatly affect the productivity of the entire process. In previous studies [9–11] it was found that, once the cells were re-supplied with nitrogen (N) after a long N-starvation period, the TAGs, which were accumulated during N-starvation, were rapidly degraded, thus drastically reducing the TAG productivity of the entire process. Cell recovery in repeated-batch cultures depends both on the microalgal species and operational conditions, such as cycle duration, amount of re-supplied nitrogen in the medium and culture fraction remaining in the reactor after harvest.

The aim of this study was to thoroughly assess whether repeated-batch TAG production represents an effective alternative to the classical batch mode for achieving higher TAG productivities. For this, the physiological response of *Nannochloropsis* sp. to nitrogen (N) starvation and N-rich medium replenishment was investigated in lab-scale batch and repeated-batch cultivations and condensed into a mechanistic model that describes photosynthesis and carbon-partitioning during N-starvation [12] and during recovery after N-rich medium replenishment in flat panel photobioreactors. The model was used to identify potential improvements for both batch and repeated-batch processes and to compare the two processes on optimized TAG yields on light (i.e., amount of TAGs produced per mol of supplied photons in the PAR range).

## Results and discussion

The model developed by [12] for batch TAG production with *Scenedesmus obliquus* in flat panel photobioreactors was further developed to describe the effect of nitrogen (N)-starvation and N-rich medium replenishment on photosynthesis and carbon partitioning in batch and repeated-batch cultivations of *Nannochloropsis* sp.. In particular, a TAG degradation mechanism was devised for repeated-batch cultivations and implemented in the model “(General model structure” section; Additional file 1).

The model consists of a photosynthesis and a carbon partitioning module (Fig. 7a). The photosynthesis module describes the photosynthetic capacity available for metabolism, based on the incident light intensity, reactor geometry, biomass concentration, and the nitrogen content of the biomass. The carbon partitioning module (Fig. 7b) describes the partitioning of the available photosynthetic capacity into the different biomass constituents, based on the nitrogen content of the biomass. For this, the photosynthetic and conversion yields are calculated with flux balance analysis (Additional file 1: Sect. S1.1.2). Finally, material balances are used to calculate the changes in biomass concentration, biomass composition, and nitrogen content of the biomass during the cultivation, using the rates derived from the carbon partitioning module.

**Fig. 1:**
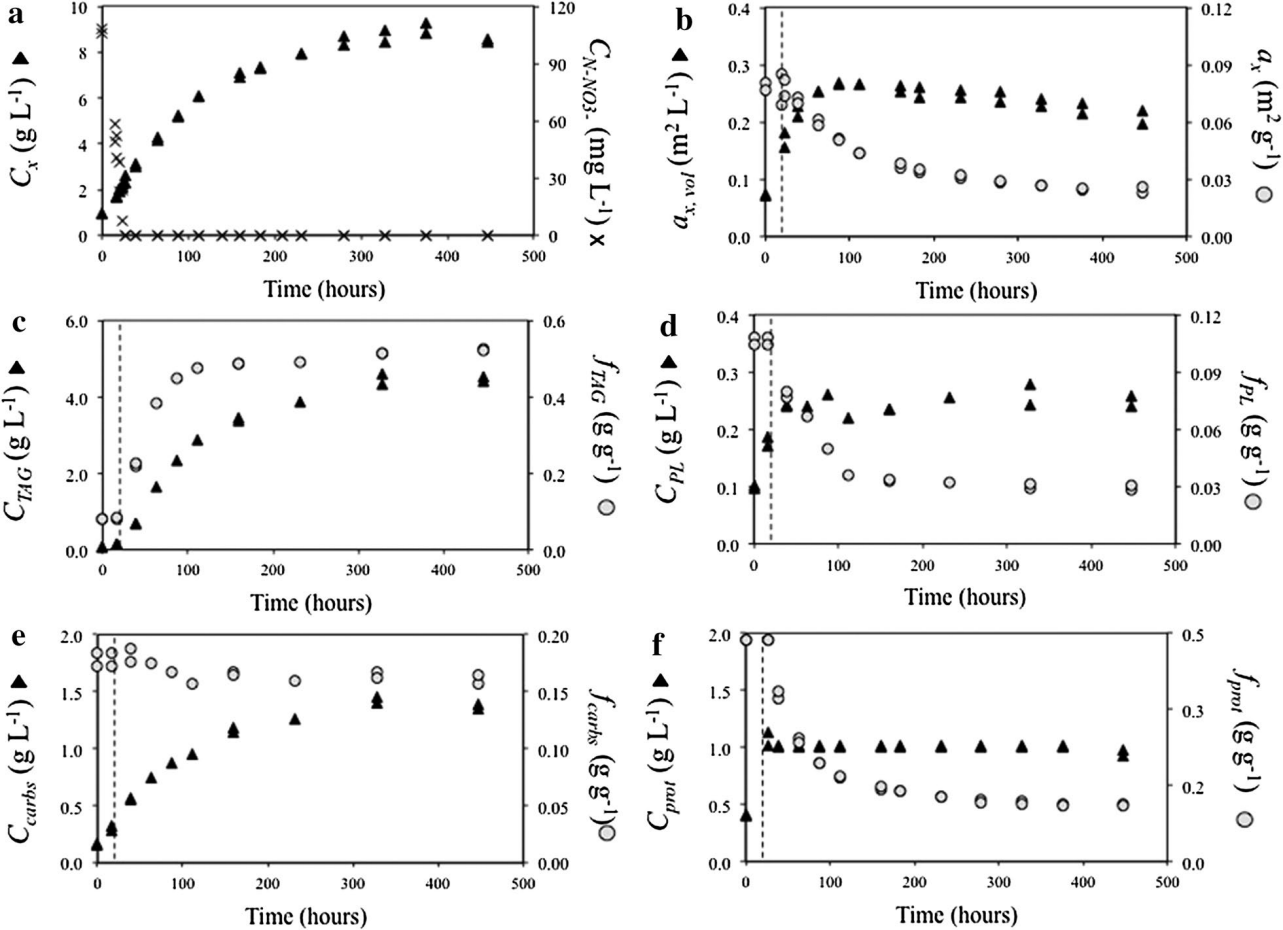
Batch nitrogen run-out cultivations. Time-evolution of **a** biomass (*C*_*x*_) and N–NO_3_^−^ (C_N_–NO_3_^−^) concentrations, **b** volumetric (*a*_*x,* vol_) and biomass-specific (*a*_*x,*_) absorption cross section, **c** TAG concentration (C_TAG_) and content (*f*_TAG_), **d** polar lipid concentration (*C*_*PL*_) and content (*f*_PL_), **e** carbohydrate concentration (*C*_carbs_) and content (*f*_carbs_), **f** estimated protein concentration (*C*_prot_) and content (*f*_prot_). The *dotted line* indicates the time point at which extracellular N-NO_3_^−^ concentration was zero. Data points for each of the duplicate cultivations are reported, indicating a very high degree of reproducibility

**Fig. 2:**
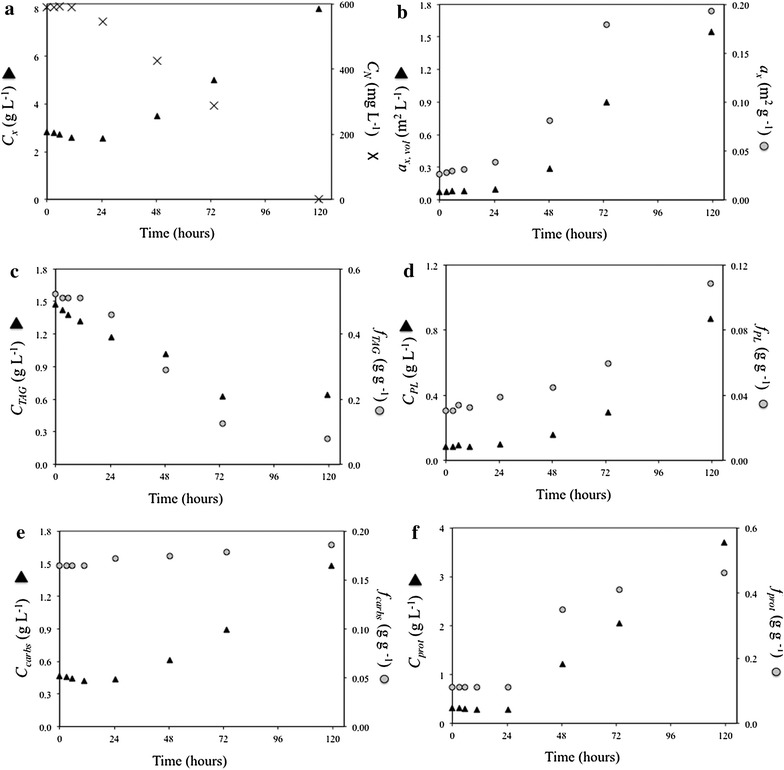
N-rich medium-replenished batch cultivation. Time-evolution of **a** biomass (*C*_*x*_) and N–NO_3_^−^ (C_N_–NO_3_^−^) concentrations, **b** volumetric (*a*_*x,* vol_) and biomass-specific (*a*_*x,*_) absorption cross section, **c** TAG concentration (C_TAG_) and content (*f*_TAG_), **d** polar lipid concentration (*C*_*PL*_) and content (*f*_PL_), **e** carbohydrate concentration (*C*_carbs_) and content (*f*_carbs_), **f** estimated protein concentration (*C*_prot_) and content (*f*_prot_)

**Fig. 3:**
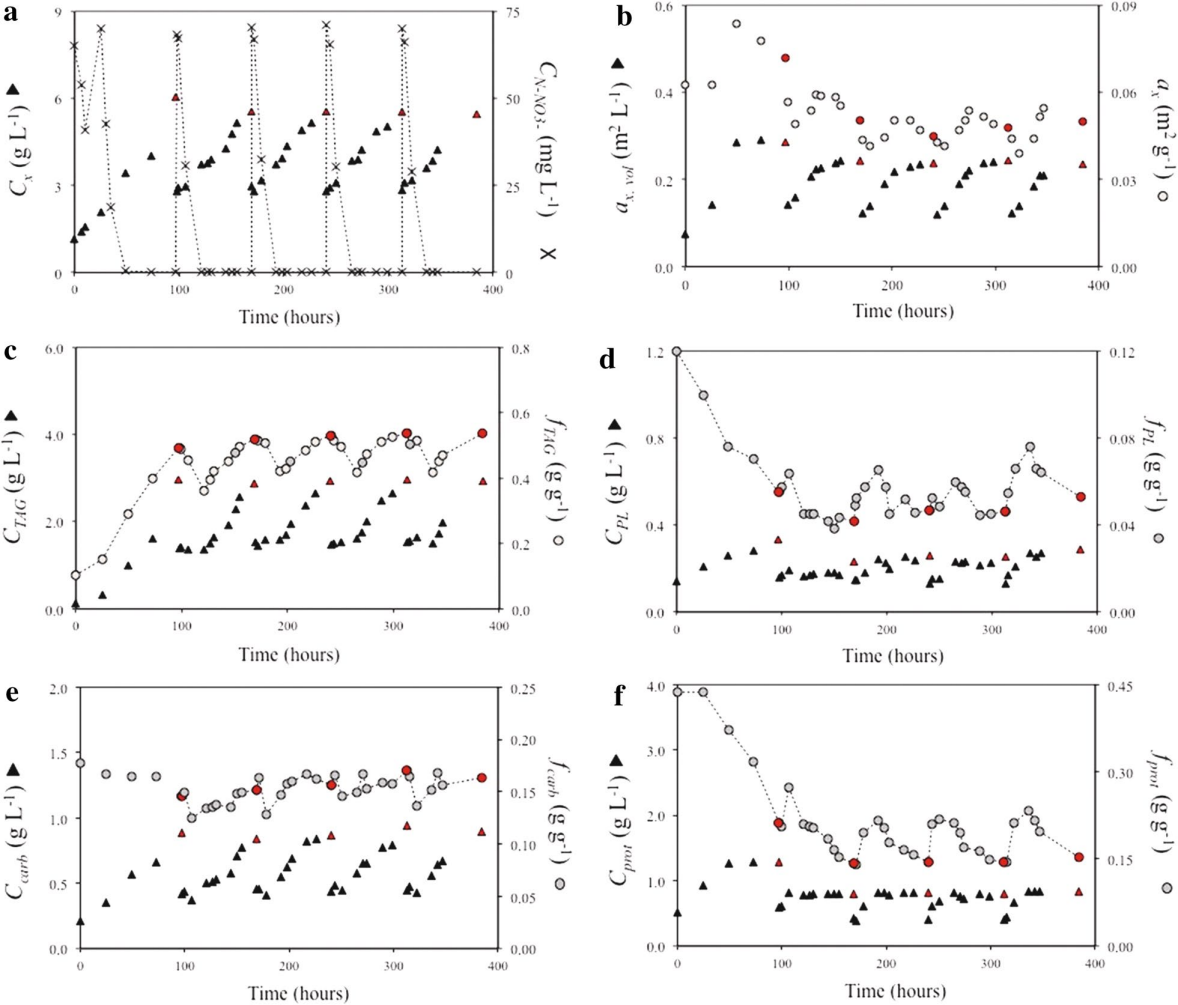
70 mg_N_ L^−1^-rich medium repeated-batch cultivation (70N). Time-evolution of **a** biomass (*C*_*x*_) and N–NO_3_^−^ (C_N_–NO_3_^−^) concentrations, **b** volumetric (*a*_*x,* vol_) and biomass-specific (*a*_*x,*_) absorption cross section, **c** TAG concentration (C_TAG_) and content (*f*_TAG_), **d** polar lipid concentration (*C*_PL_) and content (*f*_PL_), **e** carbohydrate concentration (*C*_carbs_) and content (*f*_carbs_), **f** estimated protein concentration (*C*_prot_) and content (*f*_prot_). *Red symbols* indicate the moment at which a harvest was applied. *Lines* are drawn only for illustrative purposes

**Fig. 4:**
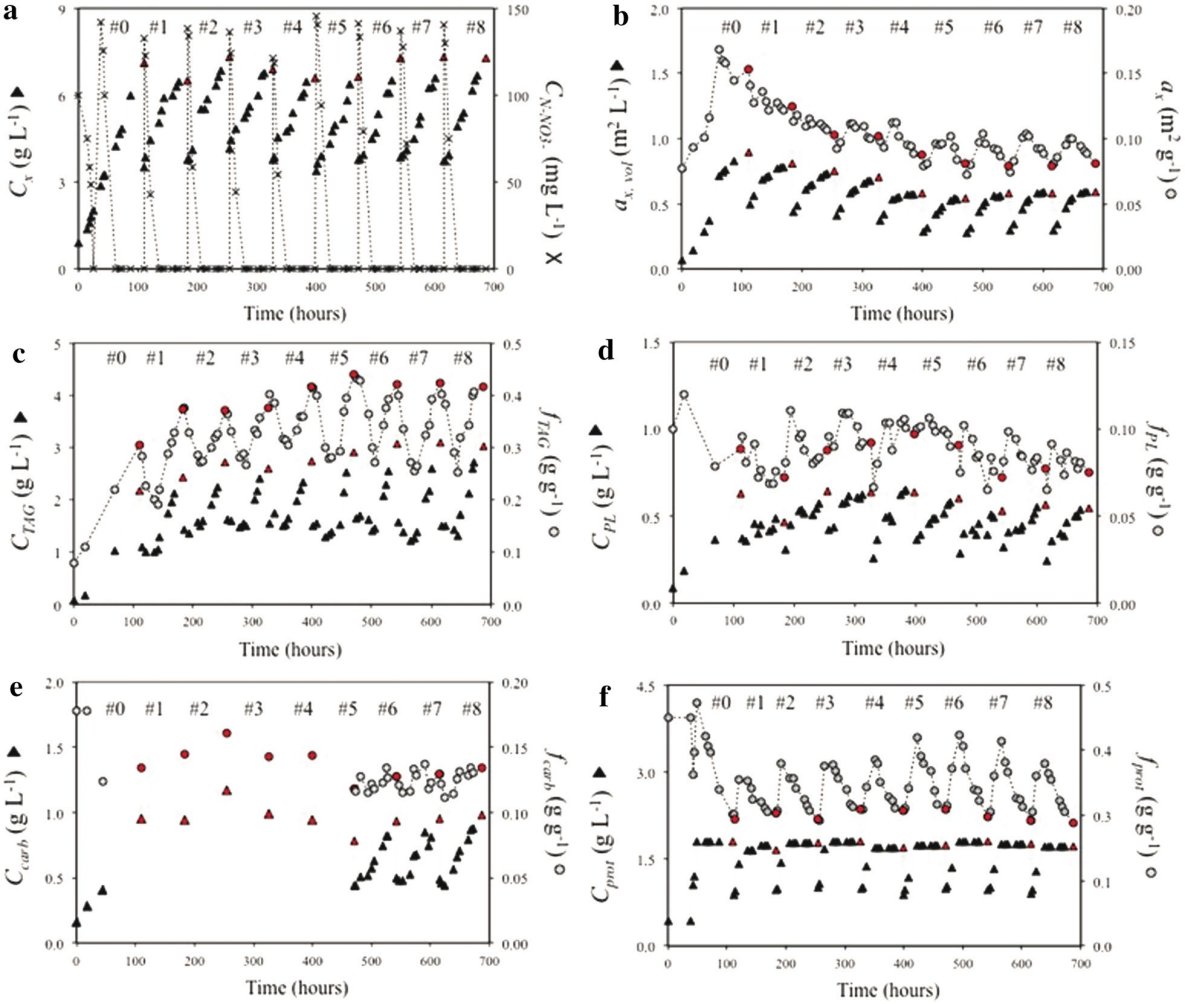
140 mg_N_ L^−1^-rich medium repeated-batch cultivation (140N). Time-evolution of **a** biomass (*C*_*x*_) and N–NO_3_^−^ (C_N_–NO_3_^−^) concentrations, **b** volumetric (*a*_*x,* vol_) and biomass-specific (*a*_*x,*_) absorption cross section, **c** TAG concentration (C_TAG_) and content (*f*_TAG_), **d** polar lipid concentration (*C*_PL_) and content (*f*_PL_), **e** carbohydrate concentration (*C*_carbs_) and content (*f*_carbs_), **f** estimated protein concentration (*C*_prot_) and content (*f*_prot_). *Red symbols* indicate the day at which a harvest was applied. *Lines* are drawn only for illustrative purposes

A nitrogen (N) run-out batch cultivation (Fig. 1) was performed to derive the parameters for initial model calibration (Additional file 1: Sect. S1.1.2). Next, N-rich medium-replenished batch (Fig. 2) and N-rich medium-replenished repeated-batch (Figs. 3, 4) cultivations were performed to further develop and validate the model for repeated-batch TAG production (Additional file 1: Sect. S1.3). The model was then used to identify potential for improvement of TAG yield on light, for both batch and repeated-batch processes, by performing Monte-Carlo sampled combinations of various biological and cultivation parameters of the model (Table 3; Fig. 6). Finally, batch and repeated-batch TAG production strategies were compared based on these optimized TAG yields on light (Table 2).

### Nitrogen run-out batch cultivation

In the batch cultivations, nitrogen (N) was depleted at a biomass concentration of about 2.45 g L^−1^ (Fig. 1a). At N-depletion, the cultures were supplied with a N-free stock to prevent other nutrients limitation, and subsequently cultured for 17 days. At the end of the cultivation, a 3.5-fold-increase in biomass concentration was observed. Although the biomass-specific absorption cross section (*a*_*x*_) showed a sudden decrease after the onset of N-starvation (Fig. 1b), the volumetric absorption cross section (*a*_*x,* vol_) increased for about 75 h from the onset of N-starvation, suggesting that, during that period, pigment synthesis continued before declining during N-starvation.

TAG concentration increased linearly during the first 120 h of N-starvation (Fig. 1c) and, within the first 75 h of N-starvation, TAGs already represented 45 % of cellular dry weight. This resulted in a maximum (time-averaged) TAG yield on light of 0.21 g mol_ph_^−1^ (calculated as described in Additional file 1: Sect. S1.1.4).

Contrarily to TAGs, estimated protein concentration did not increase from the onset of N-starvation, whereas polar lipid production ceased after 75 h. However, no net polar lipid and protein degradation occurred, as their concentration remained more or less constant until the end of the cultivation, while their content progressively decreased from 0.10 to 0.03 g g^−1^ and from 0.44 to 0.12 g g^−1^, respectively (Fig. 1d, f). From the onset of N-starvation, carbohydrate concentration increased more or less proportionally to the increase in TAGs. Hence, carbohydrate content showed only a minor decrease over time (Fig. 1e).

### Batch nitrogen-rich medium-replenished cultivation

To study the dynamics of cell recovery after a prolonged nitrogen (N)-starvation period, 700 mL of N-rich medium was resupplied to 1200 mL of the N-starved culture leading to a final N–NO_3_^−^ concentration of 590 mg L^−1^ and to a biomass concentration of 2.81 g L^−1^. No N-NO_3_^−^ uptake was observed for the first 24 h after replenishment (Fig. 2a). During that period, biomass and TAG concentrations decreased from 2.81 to 2.55 g L^−1^ and from 1.47 to 1.17 g L^−1^, respectively, with TAG content declining from 0.52 to 0.46 g g^−1^. Subsequently, within the next 96 h, N–NO_3_^−^ was completely consumed concurrently with an increase in biomass concentration (Fig. 2a), absorption cross section (Fig. 2b), as well as polar lipid (Fig. 2d), carbohydrate (Fig. 2e) and estimated protein (Fig. 2f) concentrations. Inversely, TAG concentration continued decreasing until 72 h from nutrient-replenishment and TAG content returned to basal-levels (0.08 g g^−1^) (Fig. 2c).

### Repeated-batch cultivations

Two different nitrogen (N) resupply regimes were applied in the repeated-batch cultivations. Every 72 h, 50 % of the culture volume was harvested while the remaining fraction was re-supplied with N-rich medium such that the final N–NO_3_^−^ concentration in the reactor was either 70 mg L^−1^ (70N) or 140 mg L^−1^ (140N). The repeated-batch cultivations were stopped when three consecutive and constant cycle repetitions (i.e., steady-state cycles) were achieved (cycles #2–4 for 70N, and cycles #6–8 for 140N) (Figs. 3, 4; Additional file 2). At the harvest of the constant cycle repetitions, biomass, TAG, estimated protein and carbohydrate concentrations, pigmentation as well as biomass-specific TAG production rates and nitrogen consumption rates were equal for the consecutive steady-state cycles (standard deviation within 5 % of average). The following sections and discussion will primarily focus on the constant cycle repetitions.

Overall, biomass concentration was higher for 140N than for 70N. In the constant cycle repetitions, biomass concentration increased from 2.88 ± 0.10 to 5.52 ± 0.05 g L^−1^ for 70N (Fig. 3a) and from 3.78 ± 0.07 to 7.30 ± 0.01 g L^−1^ for 140N (Fig. 4a). The volumetric absorption cross section (*a*_*x,* vol_) increased until the end of the cycle for 140N, and only for the first 34 h of the cycle for 70N, suggesting net pigment production during those periods. Contrarily, a sudden decrease in biomass-specific absorption cross section (*a*_*x*_) was observed immediately after culture harvest and dilution for both cultivations (Figs. 3b, 4b). Then, *a*_*x*_ increased before declining again with the progression of N-starvation.

At the harvest of the constant cycle repetitions, TAG concentration was similar (2.95 ± 0.02 g L^−1^ for 70N and 3.07 ± 0.04 g L^−1^ for 140N) for the two cultivations, thus resulting in similar TAG yields on light (0.13 ± 0.01 g mol_ph_^−1^ for 70N and 0.12 ± 0.01 g mol_ph_^−1^ for 140N) (Additional file 2), whereas TAG content was 0.54 ± 0.01 g g^−1^ for 70 N and 0.42 ± 0.01 g g^−1^ for 140N. In both 70N and 140N cultures, only negligible amounts of TAGs were degraded upon N-resupply (Figs. 3c, 4c). During the first 24–30 h of the constant cycle repetitions, TAG concentration remained rather constant while the cellular TAG content decreased from 0.54 ± 0.01 to 0.42 ± 0.01 g g^−1^ (70N) and from 0.42 ± 0.01 to 0.27 ± 0.03 g g^−1^ (140N). Remarkably, in both cultures, the decrease in TAG content proceeded for about 10 h after nitrogen was depleted from the medium (Figs. 3c, 4c).

Polar lipid concentration increased until harvest in both cultivations, indicating a net production of polar lipids during the cycle, whereas polar lipid content remained rather constant (0.07–0.04 g g^−1^ for 70N and 0.11–0.07 g g^−1^ for 140N) (Figs. 3d, 4d). Carbohydrate concentration steadily increased over the cycle, indicating a net production of carbohydrates in both cultures, whereas carbohydrate content showed only minor fluctuations upon N-depletion (Figs. 3e, 4e).

Cellular nitrogen content fluctuated between 0.026 ± 0.000 and 0.038 ± 0.001 g g^−1^ (70N) and between 0.041 ± 0.001 and 0.065 ± 0.005 g g^−1^ (140N), peaking, in both cases, at 24 h of the cycle. In both cultivations, estimated protein concentration increased during the first 10–24 h after N-supply (Figs. 3f, 4f). Upon N-depletion, estimated protein concentration remained constant in both cultures.

### Physiological responses to nitrogen starvation and replenishment

#### Carbon partitioning during N-starvation

As it is reflected by the high TAG contents of early N-starvation, TAGs were produced at high rates in both nitrogen run-out batch and repeated-batch cultivations (Figs. 1c, 3c, 4c), and no other storage compound was accumulated in response to N-starvation (Figs. 1e, 3e, 4e). Differently, [13] reported for *Nannochloropsisoceanica* IMET1 during early N-starvation, a sequential expression of genes involved first in β-(1,3)-glucans (e.g., chrysolaminarin and laminarin) synthesis and then in their degradation. The authors concluded that these sugars were inter-converted into TAGs. However, as it could be deduced from the changes in biomass composition during all our experiments (Figs. 1, 2, 3, 4), this was not the case for our strain. In fact, in our cultivations, carbohydrate concentration increased proportionally to all other biomass components (i.e., polar lipids, TAGs and estimated proteins) such that their content showed only minor fluctuations. Furthermore, the increase in carbohydrate concentration was observed immediately after N-resupply, thus indicating that the carbohydrates produced during N-starvation were not degraded. Hence, it can be concluded that carbohydrates have a structural role rather than a storage function in *Nannochloropsis* sp.. In addition, as no net degradation of polar lipids was observed during our cultivations (Figs. 1d, 3d, 4d), a net conversion of polar lipids into TAGs could be excluded for *Nannochloropsis* sp., which is in contrast from what is reported for *Nannochloropsisgaditana* [14] and *Nannochloropsis oceanica* IMET1 [13].

#### Nitrogen uptake upon nitrogen-rich medium resupply

In both repeated-batch cultivations, nitrogen (N) uptake started immediately after N-rich medium resupply and so did the synthesis of polar lipids, proteins, and carbohydrates (i.e., reproducing biomass). Differently, in the N-rich medium-replenished batch cultivation no net nitrogen uptake, and thus no synthesis of reproducing biomass, was observed during the first 24 h after N-rich medium resupply (Fig. 2). This is in contrast with similar N-rich medium replenishment batch studies on *Chlorella zofingiensis* [9] and *Dunaliella tertiolecta* [15], for which an almost immediate N-uptake was observed. Besides species-specific differences, the delayed N-uptake can likely be attributed to an energy shortage to fuel N-uptake, which could be due to a low remaining photosynthetic activity caused by the much higher stress pressure to which our N-rich medium-replenished batch culture was subjected. Indeed, the combination of higher light intensity (636 vs. 150 μmol m^−2^ s^−1^ [15] or 500 μmol m^−2^ s^−1^ [9]) and longer N-starvation resulted in a severe impairment of the photosynthetic machinery as indicated by the low maximum PSII efficiency (*F*_v_*/F*_m_) at the moment of N-rich medium resupply (Additional file 3). Moreover, *F*_v_*/F*_m_ at the moment of N-rich medium resupply was substantially lower in our N-rich medium-replenished batch culture (0.20) than in our repeated-batch cultivations (0.40–0.50) for which N-uptake did commence immediately after N-rich medium resupply (Figs. 3, 4).

#### TAG degradation upon nitrogen-rich medium resupply

Net TAG degradation was observed for the nitrogen (N)-rich medium-replenished batch cultivation (Fig. 2c), whereas it was negligible in the repeated-batch cultures (Figs. 3c, 4c). In the N-rich medium-replenished batch culture, TAG degradation commenced immediately after N-rich medium replenishment, likely to generate energy and building blocks to initiate N-uptake and the recovery process. TAGs were degraded at a constant rate to baseline-levels promoting full cell recovery after 72–120 h from N-rich medium resupply. The observed TAG respiration is in line with the hypothesis that TAGs are accumulated as energy reserve to fuel nitrogen and carbon metabolism once favorable growth conditions are restored but photosynthesis alone cannot initiate recovery and reproductive processes [10, 11, 16]. For the repeated-batch cultivations, substantial TAG degradation was not observed, because, although the cells were repeatedly subjected to N-starvation cycles, they were exposed to shorter N-starvation periods. The hypothesis that TAG degradation after N-rich medium replenishment occurs only when the photosynthetic capacity is heavily impaired, is further supported by the lower *F*_v_*/F*_m_ value of the N-rich medium-replenished batch cultivation compared to the *F*_v_*/F*_m_ of the repeated-batch cultures at the moment of N-rich medium resupply (Additional file 3). Thus, in repeated-batch cultivations, TAG degradation is expected only for very high stress pressures (i.e., combinations of long cycle durations, low amounts of re-supplied nitrogen in the medium and high remaining culture fractions after harvest).

### Comparison with literature

Table 1 compares the TAG yields on light obtained in this study with previously published yields for similar batch and repeated-batch cultivations in flat panel PBRs. Note that for batch, only the N-starvation period is considered. In such a way, the effect of the different N-replete growth phases, which were performed under different and, possibly, suboptimal conditions, is neglected.

**Table 1:** Comparison with literature

Species	TAG yield (g mol_ph_^−1^)	Light intensity (μmol m^−2^ s^−1^)	Reference
Batch (nitrogen starvation phase)			
* C. zofingiensis*	0.19	500	[16]
* C. vulgaris*	0.05	270	[21]
* N. oculata*	0.17	250	[22]
* Nannochloropsis* sp.	0.14	636^a^	Benvenuti et al. (personal communication)
* Nannochloropsis* sp.	0.34	636	This study
* N. oleoabundans*	0.17	218	[23]
* N. oleoabundans*	0.03	270	[24]
* S. obliquus*	0.22	500	[25]
Starchless *S. obliquus*	0.37	500	[25]
Repeated-batch (constant cycle repetitions)			
* C.* *pyrenoidosa*	0.12	175	[26]
* Nannochloropsis* sp.	0.07	636^a^	Benvenuti et al. (personal communication)
* Nannochloropsis* sp.	0.13	636	This study (70N)
* Nannochloropsis* sp.	0.12	636	This study (140N)

Comparison of TAG yield on light obtained with various species for both lab-scale batch and repeated-batch cultivations in flat panel PBRs. The nitrogen starvation phase and the constant cycle repetitions are considered for batch and repeated-batch, respectively. 70N: 70 mg_N_ L^−1^-rich medium repeated-batch cultivation; 140N: 140 mg_N_ L^−1^-rich medium repeated-batch cultivation

^a^Average daily light intensity supplied as a day/night cycle

Despite that in the studies of [17–23] with different species, a lower incident light intensity was applied, which is a condition known to be beneficial for TAG yield on light [24], comparable or higher batch and repeated-batch TAG yields were obtained with *Nannochloropsis* sp. in the present study. This confirms that this species is a highly productive microalga [5].

The lower yields reported by Benvenuti et al. (personal communication) for lab-scale batch and repeated-batch cultivations of the same microalga subjected to day/night cycles can likely be attributed to losses due to photo-saturation at the very high light intensities experienced during the central hours of the day.

### Model simulations

**Fig. 5:**
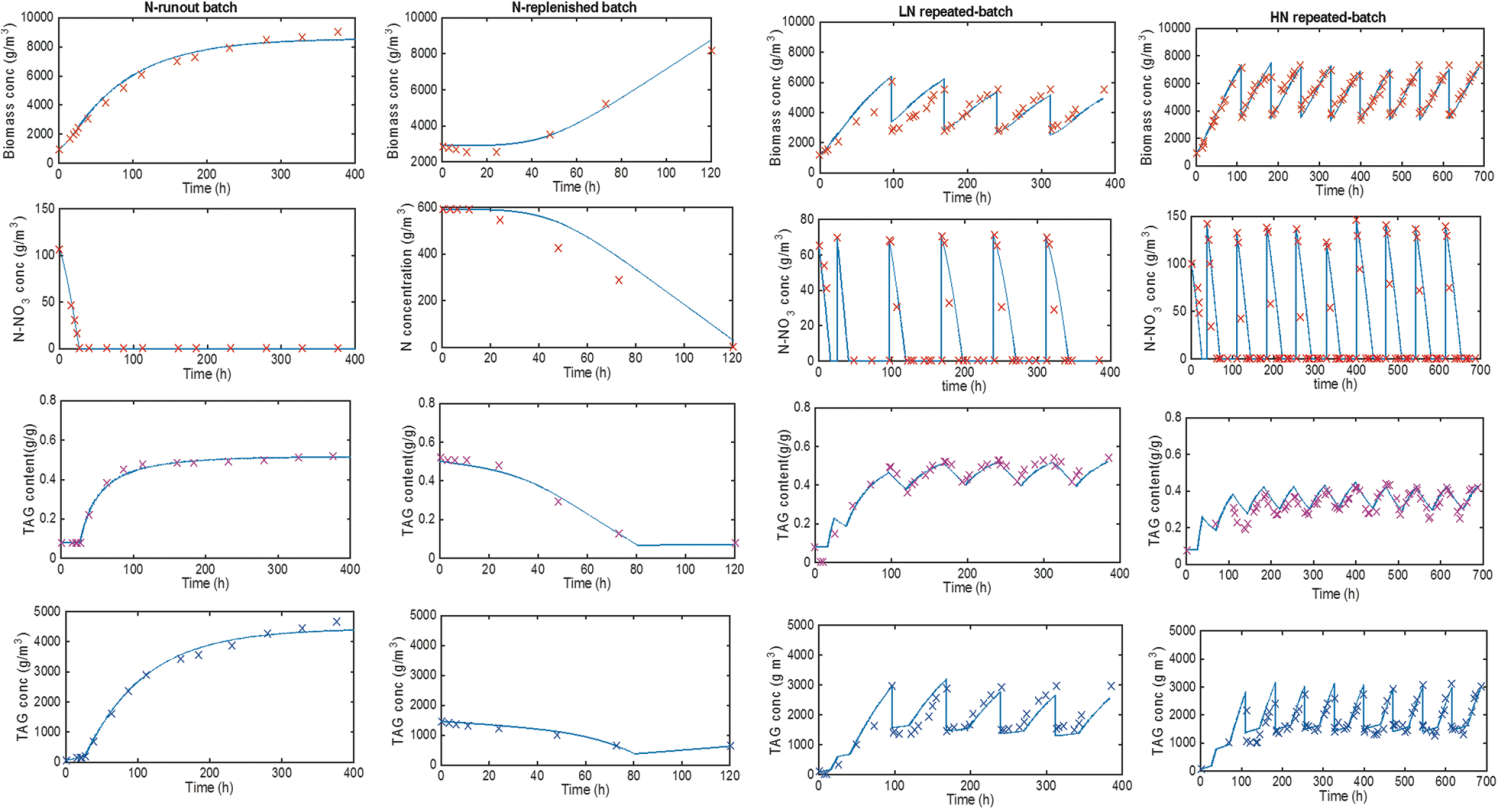
Model simulations and experimental data. Model simulations (*lines*) and experimental data (*symbols*) of biomass, external N–NO_3_^−^ and TAG concentrations as well as TAG content for the N-run-out batch, N-rich medium-replenished batch, 70 mg_N_ L^−1^-rich medium (70N) repeated-batch and 140 mg_N_ L^−1^-rich medium (140N) repeated-batch cultivations

When model simulations are performed using the parameters reported in Additional file 1: Table S1, Table S2 (Additional file 1: Sect. S1.1.2–1.3), it can be seen that the model closely follows the experimental data (Fig. 5). Furthermore, the predicted TAG yields (0.22, 0.14 and 0.15 g mol_ph_^−1^, for N-run-out batch, 70 mg_N_ L^−1^-rich medium repeated-batch [70N] and 140 mg_N_ L^−1^-rich medium repeated-batch [140N], respectively) are in close agreement with the measured TAG yields (0.21, 0.13, and 0.12 g mol_ph_^−1^, for N-run-out batch, 70N and 140N, respectively).

### Optimized TAG yield on light for batch and repeated-batch process

The model was used to identify potential for improvement of TAG yield on light for both batch and repeated-batch processes by performing Monte-Carlo sampled combinations of model parameters as reported in Table 3.

#### Effect of incident light intensity and enhanced photosynthetic machinery

In our model simulations, the incident light intensity and the maximum photosynthetic rate of nitrogen replete cells (*q*_ph_^max, replete^) were varied in order to assess the effect of these two model parameters on the photosynthetic efficiency and thus on the TAG yield on light.

**Fig. 6:**
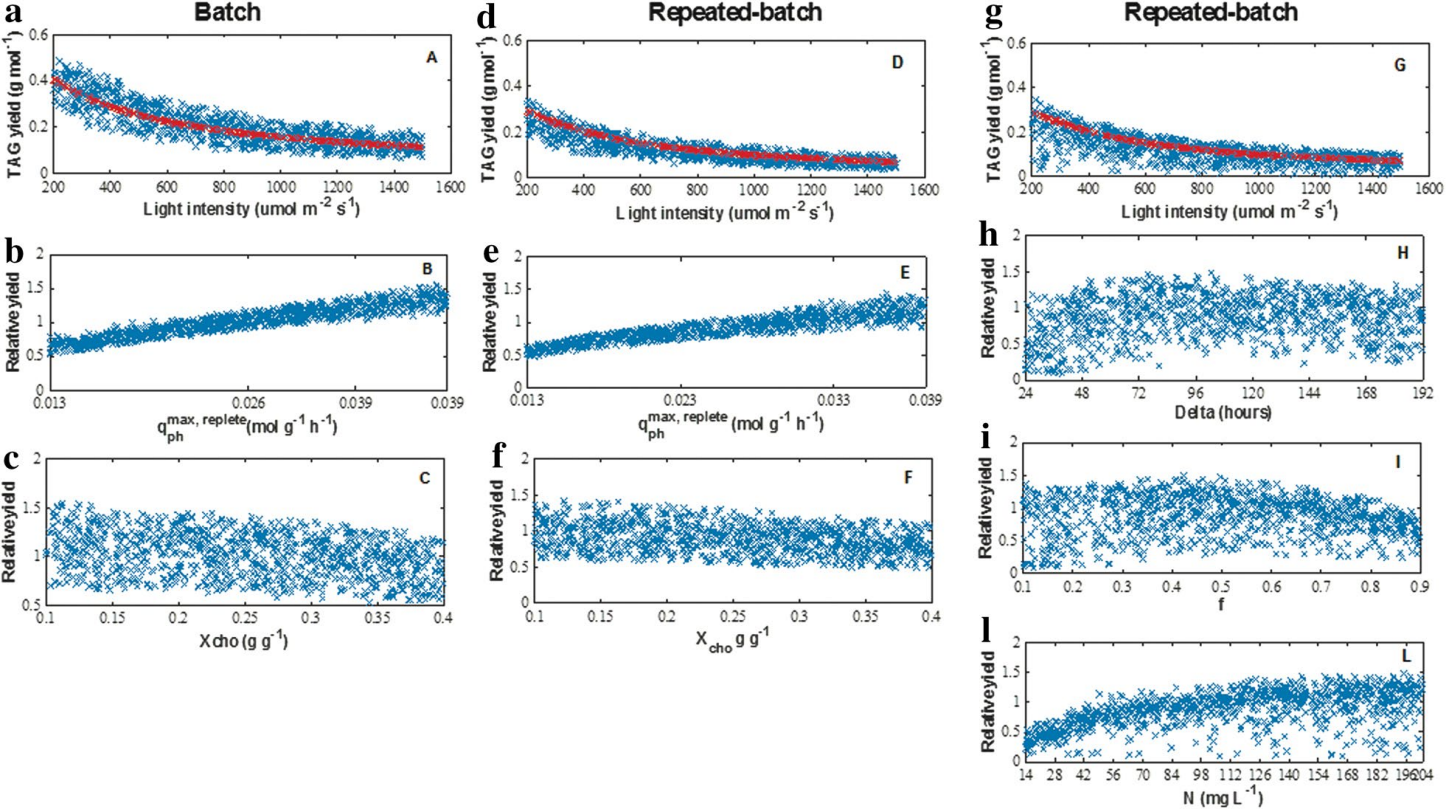
Output of Monte-Carlo-sampled simulations. **a**, **d** Impact of incident light intensity, maximum photosynthetic rate of nitrogen replete cells (*q*_ph_^max, replete^) and residual biomass made during nitrogen starvation (*X*_cho_) on batch and repeated-batch, respectively. **g** Impact of incident light intensity, cycle duration (Δ), culture fraction remaining after harvest (*f*) and amount of nitrogen resupplied in the medium (N) on repeated-batch. To illustrate the individual contribution of *q*_ph_^max, replete^ (**b**–**e**), *X*_cho_ (**c**–**f**), Δ (H), *f* (I) and N (L) independently from the influence of light, the TAG yield is normalized to the yield (*red symbols*) predicted at the same incident light intensity and with the value of the parameter under study as presented in Additional file 1: Table S1 (batch) and Table S2 (repeated-batch)

For both batch and repeated-batch, decreasing the incident light intensity had the largest positive impact on the TAG yield (Fig. 6a, d, g). For instance, in the reference case (red dots in Fig. 6), the TAG yield increased up to fourfold when the light intensity was decreased from 1500 to 200 μmol m^−2^ s^−1^ (from 0.12 to 0.41 g mol_ph_^−1^ and from 0.07 to 0.29 g mol_ph_^−1^ for batch and repeated-batch, respectively). By reducing the incident light intensity, the extent of photosaturation decreased [12, 24]. In practice, a reduction of light intensity can be partly achieved by applying the principle of light dilution using vertically oriented PBRs [25].

The maximum photosynthetic rate decreases during nitrogen (N) starvation [26] and consequently, the photosystem becomes saturated at lower light intensities (Additional file 1: S1.1.1, Eq. S1). Another approach to diminish photosaturation under N-starvation is to enhance the photosynthetic machinery by increasing the maximum photosynthetic rate under nitrogen (N) replete conditions (*q*_ph_^max, replete^) (Additional file 1: S1.1.1, Eq. S3). In our model simulations, increasing *q*_ph_^max, replete^ resulted in higher TAG yields on light (Fig. 6b, e). The largest relative improvement (32–34 %) was observed at high light intensity, where photosaturation mostly occurs, rather than at low light intensity, for which an 11–13 % relative improvement was nonetheless found.

#### Higher TAG yield on light by improved carbon partitioning

As *Nannochloropsis* sp. does not accumulate other storage compounds besides TAGs during nitrogen (N) starvation (Figs. 1, 2, 3, 4 and “Carbon partitioning during N-starvation” section), this alga already has a much more favorable carbon partitioning mechanism compared to other species [21, 23, 24]. However, a further improvement of the carbon partitioning could be achieved by decreasing the residual biomass fraction of N-starved biomass (*X*_cho_). Regardless of the incident light intensity, a lower *X*_cho_ can result in a 10–16 % relative improvement for the batch (Fig. 6c), whereas a negligible improvement was observed for the repeated-batch (Fig. 6f), which was already characterized by a relatively low *X*_cho_ compared to the batch experiment.

#### Higher TAG yield on light by optimized operational settings

The operational settings have a strong influence on the TAG yield on light for both batch and repeated-batch TAG production processes. The influence of biomass concentrations at the onset of nitrogen (N) starvation (*C*_*x, N* =_ _*0*_) and reactor light path (*z*) has been already highlighted by several authors [7, 27–30]. However, in our model simulations, almost no improvement in yield was observed compared to the reference case (Additional file 4). This can be attributed to the low maintenance coefficient (*m*_*s*_) that was used as model input. The low *m*_*s*_ limited the negative effect of the high maintenance requirements that are usually associated to long *z* and high *C*_*x, N* =_ _*0*_ [12, 31].

For repeated-batch, the effect of the amount of resupplied nitrogen in the medium (N, in mg L^−1^), cycle duration (Δ, in hours) and remaining culture fraction after harvest (*f*) was assessed. Trends for individual settings could be identified. For instance, regardless of the light intensity, short Δ (<48 h) result in a lower yield compared to the reference case (Fig. 6h), whereas longer Δ led to a 1.5-fold maximum relative improvement. Higher yields compared to the reference case could be identified when lowering *f* (Fig. 6i), whereas the opposite was found for N (Fig. 6l). For the latter case, the high yields at high N-resupply are attributed mostly to an enhanced biomass production rather than to high TAG contents.

In general, the combination of low N-supply, long cycle and high remaining culture fractions resulted in a high TAG content but severely reduced biomass production, thus causing low TAG yields on light. The same result was observed when a low stress pressure (e.g., combinations of high N-supply, short cycle and low remaining culture fraction) was applied. Highest yields were found for optimal combinations of the abovementioned settings. For instance, at low (LL: 200–300 µmol m^−2^ s^−1^), intermediate (IL: 550–650 µmol m^−2^ s^−1^) and high light (HL: 1400–1500 µmol m^−2^ s^−1^) intensities, the highest TAG yields (0.31, 0.18, and 0.10 g mol_ph_^−1^ on average, for LL, IL, and HL, respectively) were achieved with combinations of: 138 Δ, 0.34 *f*, 149N (LL); 128 Δ, 0.37 *f*, 154N (IL); 115 Δ, 0.37 *f*, 154N (HL).

#### Effect of operational settings on TAG degradation

**Fig. 7:**
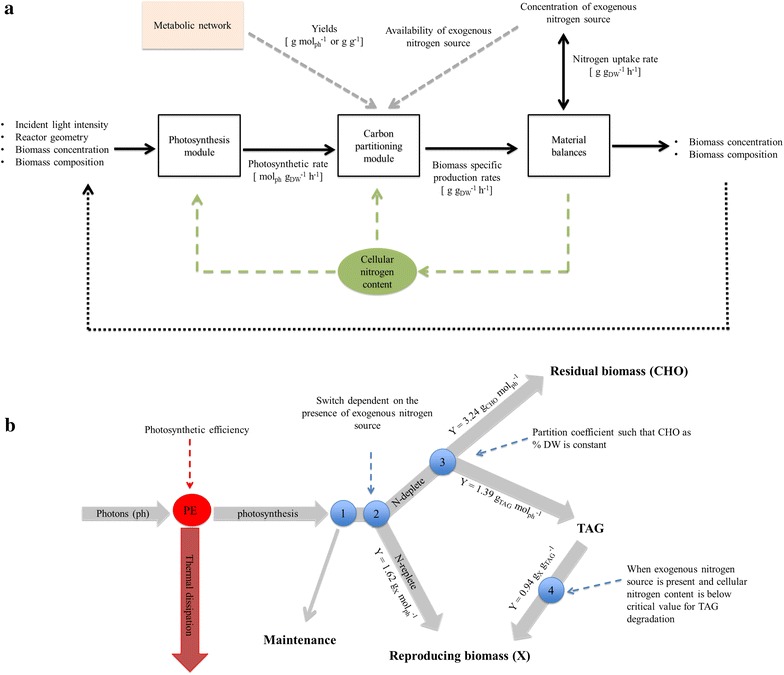
Model structure. **a** Schematic model overview. **b** Carbon partitioning module. Figure adapted from [12]

According to our hypothesis and experimental data, TAG degradation occurs only when high stress pressures are applied (Fig. 7b), namely only for combinations of long cycle durations (Δ > 72 h), low amounts of re-supplied nitrogen in the medium (N < 70 mg L^−1^) and high culture fractions remaining in the reactor after harvest (*f* > 0.5). Under such operational settings, TAG yields on light are generally much lower (0.02–0.13 g mol_ph_^−1^) compared to those obtained under optimized operational settings where no TAG degradation occurs (0.10–0.31 g mol_ph_^−1^). Nevertheless, in our model, TAG degradation has a beneficial effect on the TAG yield on light. For example, when the longest cycle (Δ = 192 h), the lowest N-supply (N = 14 mg L^−1^) and the highest remaining culture fraction (*f* = 0.9) are simulated for an incident light intensity of 636 µmol m^−2^ s^−1^, approximately 5 % of the TAGs made during the previous cycle are degraded. However, TAG yield is about 1.3-fold higher compared to the case in which operational settings and light intensity are the same but TAG degradation is switched off. This can be explained by the fact that, as a result of TAG degradation, a faster restoration of the photosynthetic capacity was obtained. This translates in a higher photosynthetic rate, faster uptake of nitrogen, larger biomass production and slightly lower TAG content. Nonetheless, it should be pointed out that the quality of the model predictions in the range of those settings for which TAG degradation is modeled to occur, depends on the validity of our hypothesis on the TAG degradation mechanism (Fig. 7b; “Carbon partitioning module and TAG degradation mechanism” section).

### Process comparison

In this section, batch and repeated-batch TAG production processes are compared on the optimized TAG yields on light (Table 2), as identified with the Monte-Carlo sampled simulations of model parameters (Table 3).

**Table 2:** Process comparison

Scenario		TAG yield on light (g mol_ph_^-1^)	TAG content (g g^-1^)
*Batch*			
1B	HL (Base case)	0.12	0.42
2B	IL	0.23	0.42
3B	LL	0.41	0.43
	*Increased maximum photosynthetic rate and decreased residual biomass fraction*		
4B	HL	0.18	0.52
5B	IL	0.31	0.52
6B	LL	0.49	0.53
*Repeated-batch*			
1RB	HL (Base case)	0.07	0.54
2RB	IL	0.15	0.52
3RB	LL	0.29	0.44
	*Optimal N-resupply, cycle duration and harvest volume*		
4RB	HL	0.09	0.50
5RB	IL	0.18	0.51
6RB	LL	0.33	0.48
	*Increased maximum photosynthetic rate and decreased residual biomass fraction*		
7RB	HL	0.10	0.60
8RB	IL	0.19	0.54
9RB	LL	0.34	0.49
	*Optimal N-resupply, cycle duration and harvest volume and Increased maximum photosynthetic rate and decreased residual biomass fraction*		
10RB	HL	0.11	0.35
11RB	IL	0.22	0.47
12RB	LL	0.39	0.48

Optimized TAG yields on light and TAG contents. *B* batch; *RB* repeated-batch. *HL* High; *IL* intermediate and *LL* low light intensities correspond to incident light intensities of 1500, 600 and 200 μmol m^−2^ s^−1^, respectively. The model parameter values at which the TAG yields on light and TAG contents were achieved, are reported in Additional file 6

**Table 3:** Parameter ranges for Monte-Carlo-sampled simulations

Monte-Carlo-sampled simulations	*I*_0_ (µmol m^−2^ s^−1^)	*z* (m)	*C*_*x, N*= *0*_ (g m^−3^)	*q*_ph_^max, replete^ (mol g^−1^ h^−1^)	*X*_cho_ (g g^−1^)	N (mg L^−1^)	*fs*	Δ (hours)
*Batch*								
Reference case	200–1500	0.02	2451	0.026	0.31	–	–	–
Variable *I*_*0*_, *C*_*x, N* = *0,*_ *z*	200–1500	0.01–0.04	1226–4903	0.026	0.31	–	–	–
Variable *I*_*0*_, *q*_ph_^max,rep*l*ete^, *X*_cho_	200–1500	0.02	2451	0.013–0.039	0.10–0.40	–	–	–
*Repeated-batch*								
Reference case	200–1500	0.02	–	0.026	0.17	70	0.5	72
Variable *I*_*0*_, N, *f*, Δ	200–1500	0.02	–	0.026	0.17	14–204	0.1–0.9	24–192
Variable *I*_*0*_, *q*_ph_^max,replete^, *X*_cho_	200–1500	0.02	–	0.013–0.039	0.10–0.40	70	0.5	72
Variable *I*_*0*_, N, *f*, Δ, *q*_ph_^max,replete^, *X*_cho_	200–1500	0.02	–	0.013–0.039	0.10–0.40	14–204	0.1–0.9	24–192

Ranges in which parameters were varied for the Monte-Carlo-sampled simulations for batch TAG production. With *I*_0_: incident light intensity; *z*: reactor light path; *C*_*x, N* = *0*_: biomass concentration at onset of nitrogen (N) starvation, *q*_ph_^max, replete^: maximum photosynthetic rate of nitrogen replete cells, *X*_cho_: residual biomass fraction made during N-starvation; N: amount of nitrogen resupplied after each harvest; *f*: remaining fraction in the reactor after harvest; Δ: cycle duration

Optimized TAG yields on light were always higher for the batch than for repeated-batch. For the batch, optimized TAG yields ranged from 0.12 g mol_ph_^−1^ (scenario 1B) to 0.49 g mol_ph_^−1^ (scenario 6B) and, at harvest, a TAG content of 0.42–0.53 g g^−1^ was obtained. For the repeated-batch, optimized TAG yields ranged from 0.07 g mol_ph_^−1^ (scenario 1RB) to 0.39 g mol_ph_^−1^ (scenario 12RB). At harvest, TAG contents of 0.35–0.60 g g^−1^ were predicted. Furthermore, as it can be deduced from Table 2, during TAG production also the non-TAG-biomass yield on light was generally higher for the batch than for the repeated-batch. Several cellular compounds contribute to the non-TAG- biomass yield on light, such as proteins, sugars, non-acyl lipids, glyco- and phospholipids [32, 33]. Therefore, provided that biorefinery of the complete biomass is pursued [34], the potential for biomass valorization for both TAGs and non-TAG compounds is better for batch than for repeated-batch.

The advantage of the batch relies on starting with a N-replete inoculum, thus with cells that have an intact photosynthetic capacity (Additional file 3). Differently, repeated-batch cycles start with N-starved cells. Likely, the reduced photosynthetic capacity of these cells leads to an inefficient use of light during the regrowth phase, thus resulting in lower TAG yields on light compared to batch processes.

Noteworthy, when correcting the TAG yields obtained at high light intensity (scenarios 1B and 1RB of Table 2) for an assumed average loss of 15 % due to night respiration [12, 35], comparable yields with those reported for outdoor batch [7, 36, 37] and repeated-batch [31, 37, Benvenuti et al. personal communication] cultivations are found.

### Outlook on future research

The model is able to describe batch and repeated-batch TAG production and it is a very useful tools for comparing different TAG production processes. Nevertheless, certain future steps need to be taken to further develop the model.

Although the experimental data (Figs. 2, 3, 4; Additional file 3) do not contradict our hypothesis regarding the TAG degradation mechanism (Fig. 7b) and the model is able to well describe TAG degradation in N-rich medium-replenished batch cultivations (Fig. 5), the dataset is not complete enough and the TAG degradation mechanism should be validated for repeated-batch operations. For this, repeated-batch cultivations in which higher stress pressures are applied (e.g., combinations of low amounts of nitrogen supplies, long cycle durations and high culture fraction remaining after harvest) should be experimentally tested.

The maintenance coefficient (m_s_) found through parameter fitting in this study (Additional file 1: Sect. S1.1.2) is lower than what is found for N-replete growth in the literature [38–40], but even if those values are used, the model is able to fit the data well (Additional file 5). Although the model seems not very sensitive to the maintenance value, it will be useful to study the maintenance requirement, and whether this is stable or changes, during N-starvation.

Finally, this study shows that under continuous light, a batch process will always result in higher TAG yields on light compared to a repeated-batch process. However, it should be emphasized that the physiological responses to N-rich medium resupply in repeated-batch processes might differ when cells are subjected to day/night cycles. By supplying the N-rich medium at night, cell recovery may take place in the dark [11] such that the daylight period can be efficiently used for TAG production. In addition to repeated-batch, batch cultures are also likely to benefit from nightly recovery. Therefore, further research under day/night cycles is necessary for a conclusive assessment.

## Conclusion

Batch and repeated-batch TAG production processes were successfully described using a mechanistic model which further allowed process comparison based on optimized TAG yields on light. According to our model simulations for continuous light, we can conclude that an optimized batch process will result in higher TAG productivities compared to an optimized repeated-batch process. This is mainly because, in repeated-batch mode, each cycle starts with nitrogen starved cells. The reduced photosynthetic capacity of these cells leads to inefficient light-use during the regrowth phase, consequently resulting in lower overall TAG yields on light compared to batch processes.

## Methods

### Growth medium

In all pre- and cultivation steps, cells were grown on disinfected and filtered natural seawater (Oosterschelde, the Netherlands; [7]) enriched with a nutrient stock consisting of (in mM final concentration): HEPES (for pre-cultivation in Erlenmeyer flasks only), 20; KH_2_PO4, 1.7; Na_2_EDTA, 0.56; FeSO_4_·7H_2_O, 0.11; MnCl_2_·2H_2_O, 0.01; ZnSO_4_·7H_2_O, 2.3·10^−3^; Co(NO_3_)_2_·6H_2_O, 0.24·10^−3^; CuSO_4_·5H_2_O, 0.1·10^−3^; Na_2_MoO_4_·2H_2_O, 1.1·10^−3^. For pre-cultivation in Erlenmeyer flasks 25 mM of NaNO_3_ was added. In the actual experiments, nitrogen was supplied as described in “Batch nitrogen run-out and repeated-batch cultivations” section.

### Batch nitrogen run-out and repeated-batch cultivations

Pre-cultures of *Nannochloropsis* sp. CCAP 211/78 were maintained in 250 mL Erlenmeyer flasks, which were placed in an orbital shaker incubator (Multitron, Infors HT, The Netherlands) at 120 rpm under 2 % CO_2_-enriched headspace, 70 % humidity. The flasks were continuously illuminated at a light intensity of 50 µmol m^−2^ s^−1^ supplied by fluorescent lamps (TL-D Reflex 36 W/840, Philips, the Netherlands). Two-week-old flask cultures were centrifuged (780 g, 5 min) to remove remaining nutrients. Subsequently, cells were re-suspended in N-rich medium such that the biomass concentration in the reactor was 0.4–0.6 g L^−1^. Cultivations were performed in a flat panel photobioreactor with a light path of 0.02 m, 1.9 L working volume and 0.08 m^2^ surface area (Labfors, Infors HT, 2010). Mass-flow controllers supplied 1.0 L min^−1^ pressurized air for mixing. The pH was set at 7.5 and controlled by means of on-demand CO_2_ addition. A culture temperature of 25 °C was maintained by water recirculation through water jackets in direct contact with the reactor cultivation chamber. Initially the ingoing light intensity was kept at 150 µmol m^−2^ d^−1^. When the biomass concentration reached 0.9–1.1 g L^−1^, the light intensity was set at 636 µmol m^−2^ d^−1^. Experiments were carried out under continuous light to isolate the effects of N-rich medium replenishment on cell recovery from those due to night respiration.

At N-depletion, the batch cultures were supplied with the N-free stock to prevent other nutrients limitation, and subsequently cultured for 17 days. In case of repeated-batch cultures, every 72 h 50 % of the culture volume was harvested, after which fresh N-rich medium was added to fill the reactor. The nitrogen source was dosed such that the final N–NO_3_^−^ concentration in the reactor at the start of each cycle was either 70 mg L^−1^ (70N) or 140 (140N) mg L^−1^.

### Batch nitrogen-rich medium-replenished cultivation

To study the dynamics of cell recovery after a prolonged nitrogen (N) starvation period, N-rich medium, containing an excess of nitrogen (i.e., 590 mg L^−1^ final N–NO_3_^−^ concentration in the reactor), was re-supplied to the batch culture. 700 mL of N-rich medium was added to 1200 mL of culture broth such that the biomass concentration at the start (*t* = 0) of the N-rich medium-replenished batch cultivation was 2.81 g L^−1^. The culture was monitored until the external N-resupply was depleted again (i.e., 120 h after N-addition).

### Offline measurements

Biomass dry weight was determined as described by [41]. The biomass content and profile of both triacylglycerols and polar lipids were analyzed as described by [42, 43]. The total carbohydrates were quantified using the method described by [44]. Cellular nitrogen content of the N-replete biomass at the start of the cultivation was measured with a Flash EA 2000 elemental analyzer (ThermoFisher Scientific, USA) at Twente University, The Netherlands. Protein content was estimated by a presumed nitrogen content in proteins of 0.16 g g^−1^ and by assuming that all nitrogen was present in proteins [43]. Residual N–NO_3_^−^ in the medium was measured with an AQ2 nutrient analyzer (Seal Analytical, USA) as described by [7]. Cellular nitrogen content throughout the cultivation period was calculated by the increase in biomass concentration, the amount of N–NO_3_^−^ consumed during the considered time period and the measured cellular nitrogen at the start of cultivation. Absorption cross section was measured as described by [41].

In general, the sum of TAG, polar lipids, carbohydrates and estimated protein mass fractions was always about 0.9 g g^−1^. Photosystem II maximum efficiency (*F*_*v*_*/F*_*m*_) was measured in a portable pulse-amplitude modulated fluorimeter (AquaPen-C AP-C 100, Photon Systems Instruments, Czech Republic; emission peak: 620 nm, saturating light pulse: 2100 µmol m^−2^ s^−1^), as described by [5].

### Modeling batch and repeated-batch TAG production

#### General model structure

The model developed by [12] for batch TAG production with *Scenedesmus obliquus* in flat panel photobioreactors was adapted to describe the effect of nitrogen (N) starvation and N-rich medium replenishment on photosynthesis and carbon partitioning in batch and repeated-batch cultivations of *Nannochloropsis* sp. and to calculate TAG yield on light as a function of the operational strategy. The model consists of a photosynthesis and a carbon partitioning module (Fig. 7a). The photosynthesis module describes the absorption of photons and the fraction of these that are actually used for photosynthesis, based on the light intensity, reactor geometry and biomass concentration (Additional file 1: Sect. S1.1.1). The carbon partitioning module (Fig. 7b; Additional file 1: Sect. S1.1.3) describes the partitioning of the available photosynthetic capacity, as calculated by the photosynthesis module, into the different biomass constituents (i.e., reproducing biomass, TAG and residual biomass). For this, the photosynthetic and conversion yields are calculated using flux balance analysis (Additional file 1: Sect. S1.1.2). Finally, material balances are used to calculate with ordinary differential equations (ODEs; Additional file 1: Sect. S1.1.3, Eqs. S12–S15) the biomass concentration and composition during the cultivation using the rates derived from the carbon partitioning module. The cellular nitrogen content is used as proxy for the extent of N-starvation and regulates both the photosynthesis and the carbon partitioning modules. The availability of exogenous nitrogen is used as a switch between metabolic processes occurring at nitrogen replete or nitrogen-depleted conditions.

##### Photosynthesis module

The photosynthesis module, as proposed by [12], was adopted without any modification to its mechanisms, as described in Additional file 1: Sect. S1.1.1. The biomass-specific photosynthetic rate at a given incident light intensity was calculated using the hyperbolic tangent equation of [45] (Additional file 1: Eq. S1) and it was defined as the rate at which photons are channeled into the electron transport chain (i.e., absorbed photons minus the dissipated photons). According to the relation described by [45], at low light intensities, the photosynthetic rate is limited by photon absorption and it increases linearly with increasing light intensity. This increase is determined by the absorption cross section and the photosynthetic quantum yield, which are both reduced during N-starvation [5, 14, 26, 46] (Additional file 1: Eqs. S2 and S4). When the light intensity increases even further, the photosystems become photosaturated and the photosynthetic rate approaches the maximum photosynthetic rate. However, under nitrogen (N) starvation, a reduction of the maximum photosynthetic rate is observed. This was described using the correlation found by [26]. According to [26] (Additional file 1: Eq. S3), the maximum photosynthetic rate decreases linearly with decreasing nitrogen content. During nitrogen starvation, this translates in lowered biomass-specific photosynthetic rates at high light intensities and an increased photosaturation. Finally, the average photosynthetic rate in the photobioreactor is calculated as the average of the local photosynthetic rates (Additional file 1: Eq. S5).

##### Carbon partitioning module and TAG degradation mechanism

The carbon partitioning mechanism (Additional file 1: Sect. S1.1.3) used by [12] for the starchless *Scenedesmus* mutant was adopted because, as it could be deduced from the changes in biomass composition observed during our cultivations, no starch or other storage metabolites are accumulated by *Nannochloropsis* sp. in response to N-starvation (Figs. 1, 3, 4; “Carbon partitioning during N-starvation” section). Furthermore, a mechanism for TAG degradation upon N-rich medium resupply was devised and implemented into the carbon partitioning module (Fig. 7b), as is described in more detail below.

The photosynthesis module describes the energy available for metabolism (“Photosynthesis module” section, Fig. 7b, node PE). This photosynthetic capacity is first used to fulfill maintenance (Fig. 7b, node 1). The latter is assumed to be proportional only to the fraction of reproducing biomass in the total biomass, and thus not to be dependent on the amount of accumulated storage metabolites. If exogenous nitrogen (N) is present, the remaining photosynthetic capacity is used to produce reproducing biomass (Fig. 7b, node 2), which is constituted of a constant ratio of proteins, carbohydrates, TAGs and other lipids (Additional file 1: Sect. S1.1.2). Under N-starvation, it is assumed that no reproducing biomass is made, but that a fraction of the remaining photosynthetic capacity is first used for the synthesis of residual biomass (CHO), consisting of structural carbohydrates, such that the CHO content in the total biomass remains constant (Fig. 7b, node 3). Finally, the remaining photosynthetic capacity is channeled into TAG synthesis. Then, a mechanism for TAG degradation upon N-rich medium resupply is devised and implemented in the model. The hypothesis is that, once N-rich medium is resupplied, TAG degradation occurs only when the photosynthetic capacity of the cells is too low to initiate recovery and reproductive processes. In the model, the intracellular nitrogen content is used as a proxy for the photosynthetic capacity. Thus, when N-rich medium is re-supplied following a N-starvation period that led to a cellular nitrogen content above a critical level (i.e., 0.025 g g^−1^, Additional file 1: Sect. S1.1.3), no TAG degradation will occur. Differently, when N-rich medium is re-supplied after a prolonged N-starvation period, during which the cellular nitrogen content has decreased below the critical level, TAGs are converted into reproducing biomass (Fig. 7b, node 4) at a fixed rate.

The photosynthetic and conversion yields as depicted in Fig. 7b are calculated using flux balance analysis (Additional file 1: Sect. S1.1.2). For modeling TAG degradation, the enzymatic reactions involved in TAG catabolism (e.g., beta-oxidation) were added to the metabolic network.

A comprehensive description of the model equations and the changes compared to the original model of [12] are reported in Additional file 1: Sect. S1.1.1.

#### Model calibration and validation

The batch model of [12] was calibrated for *Nannochloropsis* sp. using the parameter inputs derived from the nitrogen run-out batch cultivations (Fig. 1; Additional file 1: Sect. S1.1.2). Next, the physiological insights into cell dynamics upon nitrogen re-addition and recovery metabolism (e.g., TAG degradation), gathered from the repeated-batch and N-rich medium-replenished batch cultivations (Figs. 2, 3, 4), were incorporated into the calibrated model to describe repeated-batch TAG production. As described in detail in Additional file 1: Sects. S1.1.3 and S1.3, the conversion of TAGs into reproducing biomass was modeled using the critical cellular nitrogen content (0.025 g g^−1^) at which TAG degradation commences, and the TAG degradation rate (0.011 g g^−1^ h^−1^) as estimated from the N-rich medium-replenished batch cultivation.

#### Optimization of TAG yield on light

The impact of several biological and process model parameters on the TAG yield on light was investigated and potential improvements for TAG yield on light were identified. This was done performing Monte-Carlo-sampled combinations of either (1) light intensity, biomass concentration at onset of nitrogen (N) starvation and reactor light path; (2) light intensity, maximum photosynthetic rate under N-replete conditions and residual biomass fraction made during N-starvation; or (3) light intensity, cycle duration (hours), culture fraction remaining in the reactor after harvest and amount of nitrogen resupplied with the medium. These model parameters were randomly varied within the ranges shown in Table 3, after which the model was run to calculate the TAG yield on light obtained for this set of input values. As reference, these simulations were also performed using the value of the parameter under study as estimated from the experimental data (Additional file 1: Sects. S1.2, S1.3, Table S1 and Table S2).

The TAG yield on light was chosen as optimization target as this is directly related to areal TAG productivity and represents the best parameter to compare different process strategies and light intensities [12, 23]. The obtained TAG yield on light of each batch simulation corresponded to the maximum time-averaged yield found during the batch period, corrected for the inoculum production phase. For repeated-batch, the simulated yield corresponded to the yield obtained during one constant cycle repetition (Additional file 1: Sect. S1.1.4, Eqs. S20, S21).

For the batch, the ODEs, as presented in Additional file 1: Sect. S1.1.3 (Additional file 1: Eqs. S12–S15), were integrated for a time interval between 0 and 1300 h, as this was confirmed to be sufficiently large to ensure that maximum TAG yield was always achieved within that interval. For repeated-batch, 20 constant cycle repetitions were simulated. For each combination of tested parameters, 1000 iterations were performed to generate 1000 combinations of parameter values and the corresponding maximum TAG yield on light.

Finally, the optimized TAG yields on light were used to compare batch and repeated-batch on TAG production (Table 2).

## Additional files

10.1186/s13068-016-0475-4 Batch and repeated-batch mechanistic model. Comprehensive descriptions of model equations (Additional file 1: Sect. S1.1), model calibration (Additional file 1: Sect. S1.2) and model validation (Additional file 1: Sect. S1.3) procedures as well as a list of symbol (Additional file 1: Sect. S1.4) are given.
10.1186/s13068-016-0475-4 Nitrogen uptake, biomass and TAG production in repeated-batch cycles. The time at which nitrogen was depleted from the medium, the biomass-specific nitrogen uptake and TAG production rates, as well as the time-averaged biomass and TAG yields on light at the harvest of each cycle are shown for the two repeated-batch cultivations.
10.1186/s13068-016-0475-4 Maximum photosystem II efficiency in batch and repeated-batch. Time-evolution of maximum photosystem II efficiency is shown for the batch and the repeated-batch cultivations.
10.1186/s13068-016-0475-4 Effect of biomass concentration and reactor light path on batch TAG yield on light. The effect of biomass concentration at the onset of nitrogen starvation and reactor light path on the maximum TAG yield on light of batch processes is shown.
10.1186/s13068-016-0475-4 Impact of maintenance coefficient on accuracy of model predictions. The impact of the estimated maintenance coefficient on the predicted TAG yield on light is investigated and discussed.
10.1186/s13068-016-0475-4 Optimized model scenarios and corresponding parameters. Optimized TAG yields on light and TAG contents and corresponding model parameter values at which these were achieved are shown for batch and repeated-batch processes.

## Abbreviations

-*q*_N_: biomass-specific nitrogen uptake (mg g^−1^ h^−1^)
*a*_*x,* vol_: volumetric absorption cross section (m^2^ L^−1^)
*a*_*x,*_: biomass-specific absorption cross section (m^2^ g^−1^)
B: batch
carbs: carbohydrates
*C*_carbs_: carbohydrate concentration (g L^−1^)
*C*_PL_: polar lipid content (g L^−1^)
*C*_prot_: estimated protein concentration (g L^−1^)
*C*_TAG_: TAG concentration (g L^−1^)
*C*_*x, N* = *0*_: biomass concentration at onset of nitrogen starvation (g L^−1^)
*C*_*x*_: biomass concentration (g L^−1^)
*f*: remaining culture fraction in the reactor after harvest
*f*_carbs_: carbohydrate content (g g^−1^)
*f*_PL_: polar lipid content (g g^−1^)
*f*_prot_: estimated protein content (g g^−1^)
*f*_TAG_: TAG content (g g^−1^)
HL: high light
*I*_0_: incident light intensity (µmol m^−2^ s^−1^)
IL: intermediate light
LL: low light
*m*_*s*_: maintenance coefficient (mmol g^−1^ h^−1^)
N: amount of nitrogen resupplied in the medium (mg L^−1^)
ph: photon (mol)
PL: polar lipids
prot: estimated protein
*q*_ph_^ma*x,* replete^: maximum photosynthetic rate of nitrogen replete cells (mol g^−1^ h^−1^)
*q*_TAG_: biomass-specific TAG production rate (mg g^−1^ h^−1^)
RB: repeated-batch
TAG: triglyceride
*t*_*N* = 0_: time of nitrogen depletion (hours)
*x*: biomass
*X*_cho_: residual biomass made during nitrogen starvation (g g^−1^)
*Y*_*i, j*_: yield of component *i* per mole or per gram of component *j* (g mol^−1^ or g g^−1^)
*Y*_TAG, ph, HARV, CYCLE_ (*t*): time-averaged TAG yield per mol PAR photon (or TAG yield on light) at the harvest of each cycle (g mol^−1^)
*Y*_*x,* ph, HARV, CYCLE_ (*t*): time-averaged biomass yield per mol PAR photon (or biomass yield on light) at the harvest of each cycle (g mol^−1^)
*z*: reactor light path (m)
Δ: cycle duration (hours)
