# Supplementary material for: Microalgal TAG production strategies: why batch beats repeated-batch
Source: Biotechnol Biofuels. 2016 Mar 16;9:64. doi: 10.1186/s13068-016-0475-4 (PMC4793540; doi:10.1186/s13068-016-0475-4)
Supplement: Supplementary file 1 — 10.1186/s13068-016-0475-4 Batch and repeated-batch mechanistic model. Comprehensive descriptions of model equations (Additional file 1: Sect. S1.1), model calibration (Additional file 1: Sect. S1.2) and model validation (Additional file 1: Sect. S1.3) procedures as well as a list of symbol (Additional file 1: Sect. S1.4) are given. [file 13068_2016_475_MOESM1_ESM.docx]

**Additional file 1 Batch and repeated-batch mechanistic model**

The model developed by [1] for batch TAG production with *Scenedesmus obliquus* in flat panel photobioreactors was further developed to describe the effect of nitrogen (N)-starvation and N-rich medium replenishment on photosynthesis and carbon partitioning in batch and repeated-batch cultivations of *Nannochloropsis* sp.. In particular, a TAG degradation mechanism was devised for repeated-batch cultivations and implemented in the model. The following sections describe in detail the modifications made compared to the original model of [1].

**1.1 Model equations**

*1.1.1 Photosynthesis module*

The equations used by [1] for the photosynthesis module were adopted without any modification to its mechanism. These equations are listed below. For discussion of underlying assumptions, we refer to [1].

The biomass specific photosynthetic rate (*q_ph_*) at a given incident light intensity *I* was calculated using the hyperbolic tangent equation of [2] (Eq. S1).

$\text{q}\text{ph}\text{ }\text{= }\text{q}\text{ph}\text{max}\text{ tanh }\left( \frac{\text{aψ}\text{N}\text{I}}{\text{q}\text{ph}\text{max}} \right)$ Eq. S1

Where *Ψ_N_* is the photosynthetic quantum yield, *a* is the absorption cross-section and *q_ph_^max^* is the maximum photosynthetic rate. The three parameters are affected by nitrogen (N) starvation and thus, they vary during a N-starved cultivation (Eq. S2 – S4).

A linear relation between the absorption cross-section and the cellular nitrogen content (*Q*) was found also for *Nannochloropsis* sp. (Fig. S1.1). Therefore, Equation S2, as proposed by [1] could be adopted.

$\text{a}\text{ }\text{= }\text{a}\text{replete}\text{ }\frac{\text{Q}}{\text{Q}\text{max}}\text{ }$ Eq. S2

Where *a_replete_* and *Q_max_* are the biomass-specific absorption cross-section and the cellular nitrogen content of N-replete biomass, respectively.


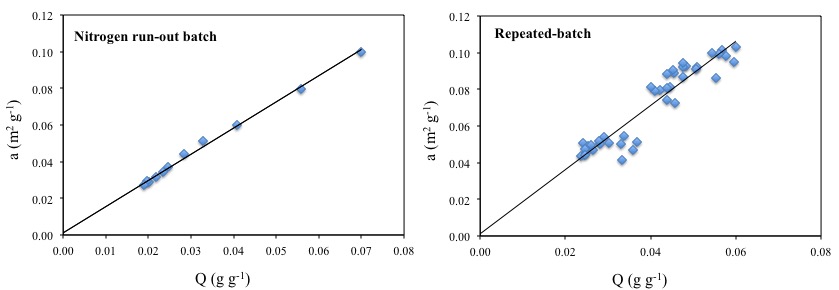


**Figure S1.1** Linear relation between absorption cross-section (*a*) and cellular nitrogen content (*Q*) for the nitrogen run-out batch and repeated-batch cultivations.

The maximum photosynthetic rate decreases linearly with decreasing nitrogen content reaching zero at the minimum nitrogen content (*Q_min_*). To describe this phenomenon, the equation proposed by [3] was adopted (Eq. S3).

$\text{q}\text{ph}\text{max}\text{ }\text{= q}\text{ph}\text{max, replete}\text{ }\left( \frac{\text{Q - Q}\text{min}}{\text{Q}\text{max }\text{-}\text{ }\text{Q}\text{min}} \right)$ Eq. S3

Where q_ph_^max, replete^ is the maximum photosynthetic rate under nitrogen replete conditions.

Furthermore, N-starvation results in a reduction of the photosynthetic quantum yield [4]. The physiological changes that determine a reduced photosynthetic quantum yield are lumped together in *Ψ_N_*, assuming that *Ψ_N_* decreases with decreasing cellular nitrogen content following a modified Droop equation [5] (Eq. S4).

$\text{ψ}\text{N}\text{ }\text{= }\left( \text{1- }\frac{\text{Q}\text{min}}{\text{Q}} \right)\left( \text{1- }\frac{\text{Q}\text{min}}{\text{Q}\text{max}} \right)^{-1}$ Eq. S4

A light gradient is present in photobioreactors, thus cells are exposed to different (high and low) local light intensities due to culture mixing. When assumed that the characteristic times of photosynthesis (1 – 10 ms, [6]) are much smaller than mixing times (order of seconds), the photosynthetic rate depends on the local light intensities throughout the photobioreactor. Therefore, the average photosynthetic rate ($\bar{\text{q}\text{ph}}$) for a flat panel photobioreactor is described by Equation S5, which neglects the effect of light scattering. Furthermore, it is assumed that the light is parallel (not diffuse) and enters the photobioreactor perpendicularly to its surface.

$\bar{\text{q}\text{ph}}\text{ }\text{= }\frac{\text{1}}{\text{z}}\int_{\text{0}}^{\text{z}} \text{q}\text{ph}\text{max}\text{ tanh }\left( \frac{\text{aψ}\text{N}\text{I}\text{0}\text{e}^{\text{(-}\text{aC}\text{x}\text{z}\text{)}}}{\text{q}\text{ph}\text{max}} \right)\text{dz}$ Eq. S5

Where *z* is the reactor light path, *I_0_* is the incident light intensity and *C_x_* is the biomass concentration.

*1.1.2 Calculation of photosynthetic and inter-conversion yields using flux balance analysis*

The theoretical maximum photosynthetic and conversion yields, as depicted in Fig. 7B, were calculated using flux balance analysis (MATLAB: *linprog*) (Eq. S6). For this, the metabolic network as described by [1] for the *Scenedesmus* starchless mutant was adopted with some modifications, which are listed below.

1. Based on our observations, reproducing biomass consists, on average, of 45% protein, 0.2% DNA, 6% RNA, 8% TAG (containing three palmitic acid molecules), 20% carbohydrates, 13.8% membrane lipids (considered as monogalactosyl-diacylglycerol molecules) and 7% ash.
2. It is assumed that all fatty acids in TAGs are palmitic acid (C16:0) molecules instead of C18:1 as presumed by [1] for *Scenedesmus*. C16:0 is indeed the most abundant fatty acid in *Nannochloropsis* sp. [4]. Due to chemical differences between these fatty acids, a slightly higher theoretical TAG yield on light is obtained for *Nannochloropsis* sp. (1.39 g mol_ph_^-1^) compared to *Scenedesmus obliquus* (1.33 g mol_ph_^-1^).
3. In our repeated-batch model, the conversion of TAGs into reproducing biomass was included. Therefore, the metabolic reactions of TAG activation, hydrolysis and oxidation were added to the metabolic network. The oxidation of C16:0 produces 8 AcCoA, 7 NADH and 7 FADH_2_. In our modified network, FADH_2_ was converted into NADH at the expenses of ATP (1 ATP per NADH). The AcCoA produced by the beta-oxidation can either be oxidized in the citric acid cycle to produce ATP and NADH, or it can be used in the glyoxylate cycle to produce malate. Both pathways were already present in the network model so no extra reactions were added to the metabolic network.

Flux balance analysis was used to determine the flux distribution that results in the highest yield on photons for each of the biomass compounds (MATLAB: *linprog*) (Eq. S6).

Objective: Maximize V_M_

Constrained with Eq. S6

S · v = 0 (stoichiometric constraints)

v_photon_ = 1 (all rates are normalized to the photosynthetic rate)

v_min_ ≤ v ≤ v_max_ (flux constraints used to describe reversibility of reactions)

Where *S* is the stoichiometric matrix and *v* is the vector containing the flux rates. The boundaries of the flux rates were set according to reversibility of reactions as described by [7]. To calculate the yield of reproducing biomass (*X*) on TAG, v_TAG_ is set to -1, v_photon_ is set to 0, v_x_ is maximized and the conversion yield is calculated as v_X_/-v_TAG_.

This procedure results in theoretical maximum yields of 1.62 g X/mol_ph_ (*Y_x, ph_*) (for growth on nitrate), 3.24 g CHO/mol_ph_ (*Y_CHO, ph_*), 1.39 g TAG/mol_ph_ (*Y_TAG, ph_*) and 0.94 gX/g TAG (*Y_x, TAG_*).

*1.1.3 Carbon partitioning module*

It is assumed that when the exogenous nitrogen concentration (*N*) is above 0, the remaining photosynthetic capacity that is not used to fulfill maintenance requirements (*m_s_*) is used for the synthesis of reproducing biomass (*X*). When *N* is zero, the synthesis of *X* is completely impaired (Eq. S7) and the remaining photosynthetic capacity is first used for the synthesis of CHO, such that CHO content in the biomass remains constant (Eq. S9), as also observed in our cultivations (Fig. 1, 3 and 4, section 2.4.1). Finally, the remainder is channeled towards TAG synthesis (Eq. S10).

A mechanism describing TAG degradation upon N-rich medium resupply is also considered. It is assumed that, when N-rich medium is resupplied after a N-starvation period such that the cellular nitrogen content is above a critical level (i.e. *Q_deg_* = 0.025 g g^-1^), no TAG degradation will occur. Differently, when N-rich medium is re-supplied after a prolonged N-starvation period, during which the cellular nitrogen content has decreased below *Q_deg_*, TAGs are converted into reproducing biomass (*X*) and TAG degradation follows 0^th^ order kinetics at the rate (*r_TAG, x_^max^*) observed in our nitrogen replenished batch cultivation (Eq. S7).

$\text{r}\text{TAG}\text{, x}\text{ = }\left\{ \begin{aligned} \text{r}\text{TAG, x}\text{max}\text{ if Q≤ Q}\text{deg}\text{ and N >0 and TAG >0} \\ \text{0 if Q}\text{ }\text{>Q}\text{deg}\text{ or N≤ 0 or TAG≤ 0} \end{aligned} \right\}$ Eq. S7

To also describe conversion of TAGs into *X*, the biomass-specific production rates of reproducing biomass (*q_x_*) and TAGs (*q_TAG_*) were redefined as shown in Equations S8 and S10, respectively.

$\text{q}\text{x}\text{ }\text{= }\left\{ \begin{aligned} \text{Y}\text{x, ph }\left( \bar{\text{q}\text{ph}}\text{ - m}\text{s}\text{ }\frac{\text{Q}}{\text{Q}\text{max}} \right)\text{+}\overset{\text{Conversion of TAG to biomass}}{\overbrace{\text{r}_{\text{TAG, x}}\text{∙ Y}_{\text{x, TAG }}}}\text{if N > 0 } \\ \text{ 0 if N≤ 0} \end{aligned} \right\}$ Eq. S8

$\text{q}_{\text{CHO}}\text{=}\left\{ \begin{matrix} \text{0} & \text{if} & \frac{\text{CHO}}{\text{c}_{\text{x}}}\text{ >}\text{X}_{\text{CHO}}\text{ or N>0} \\ \overset{\text{Available photons}}{\overbrace{\text{(}\overline{\text{q}_{\text{ph}}}\text{-}\text{m}_{\text{s}}\frac{\text{Q}}{\text{Q}_{\text{max}}}\text{)}}}\text{∙}\overset{\text{De novo CHO production}}{\overbrace{\text{Y}_{\text{CHO,ph}}}} & \text{if} & \frac{\text{CHO}}{\text{c}_{\text{x}}}\text{≤}\text{ X}_{\text{CHO}}\text{ and }\text{N≤ 0} \end{matrix} \right\}$ Eq. S9

$\text{q}_{\text{TAG}}\text{=}\left\{ \begin{matrix} \text{0} & \text{if} & \text{N}\text{ }\text{>}\text{0} \\ \left( \overset{\text{Available photons}}{\overbrace{\overline{\text{q}_{\text{ph}}\text{ }}\text{-}\text{ m}_{\text{s}}\frac{\text{Q}}{\text{Q}_{\text{max}}}}}\text{ - }\overset{\begin{aligned} \text{Photons remaining } \\ \text{after CHO production} \end{aligned}}{\overbrace{\frac{\text{q}_{\text{CHO}}}{\text{Y}_{\text{CHO, ph}}}}} \right)\text{∙}\overset{\begin{aligned} \text{De novo } \\ \text{TAG production} \end{aligned}}{\overbrace{\text{Y}_{\text{TAG, ph}}}}\text{-}\overset{\text{TAG degradation}}{\overbrace{\text{r}_{\text{TAG, x}}}} & \text{if} & \text{N≤ }\text{0} \end{matrix} \right\}$ Eq. S10

As nitrogen is present only in reproducing biomass (*X*), the biomass-specific nitrogen uptake rate (*q_N_*) depends on the production rate of *X* and its nitrogen content (*Q_max_*), resulting in Equation S11.

qN = -q_x_Q_max_ Eq. S11

Ordinary differential equations (ODEs) were adopted to describe change in reproducing biomass (*X*), *CHO*, *TAG* and exogenous nitrogen concentration (*N*) (Eq. S12 – S15). ODEs were integrated as functions of time using the 4^th^/5^th^ order Runge-Kutta algorithm by means of the MATLAB *ode45* function.

$\frac{\text{dX}}{\text{dt}}\text{= }\text{q}\text{x}\text{C}\text{x}$ Eq. S12

$\frac{\text{dTAG}}{\text{dt}}\text{= }\text{q}\text{TAG}\text{C}\text{x}$ Eq. S13

$\frac{\text{dCHO}}{\text{dt}}\text{= }\text{q}\text{CHO}\text{C}\text{x}$ Eq. S14

$\frac{\text{dN}}{\text{dt}}\text{= }\text{q}\text{N}\text{C}\text{x}$ Eq. S15

The total biomass concentration (*C_x_*) is calculated as shown in Equation S16.

C_x_ = X + CHO + TAG Eq. S16

The cellular nitrogen content of total biomass is calculated according to Equation S17.

$\text{Q = }\frac{\text{Q}\text{max}\text{ ∙ X}}{\text{C}\text{x}}$ Eq. S17

As *Nannochloropsis* sp. does not accumulate other metabolites besides TAGs during N-starvation (Fig. 1 Fig. 3 – 4 and section 2.4.1), the carbon partitioning mechanism used by [1] for the starchless *Scenedesmus* mutant was adopted, therefore no conversion of starch (or any other compound) into TAGs was considered.

Moreover, because *Nannochloropsis* sp., in contrast to *Scenedesmus obliquus*, contains considerable amounts of TAG (0.08 g g^-1^) in the N-replete reproducing biomass, the TAG content during N-starvation was corrected for the TAGs present in the reproducing biomass (*X_TAG, x_*), according to Equation S18.

$\text{TAG content =}\frac{\text{ X}\text{TAG, x }\text{∙ X+}\text{ }\text{TAG}}{\text{C}\text{x}}$ Eq. S18

CHO content was instead calculated as proposed by [1], resulting in Equations S19.

$\text{CHO content = }\frac{\text{X}\text{CHO, x}\text{ ∙ X + CHO}}{\text{C}\text{x}}$ Eq. S19

Where *X_CHO, x_* is the CHO fraction in reproducing biomass and assumed to be 0.31 g g^-1^ for batch (Table S1) and 0.17 g g^-1^ for repeated-batch (Table S2).

*1.1.4 Calculation of projected TAG yield on light for batch and repeated-batch*

Equation S20 was used to calculate projected TAG yields on light (*Y_TAG, ph_*). For the batch, the TAG yield is the maximum time-averaged yield, whereas for the repeated-batch, it is the yield at the harvest of the constant cycle repetitions.

To perform a fair comparison between batch and repeated-batch strategies, *Y_TAG, ph_* of the batch was corrected the inoculum production. Therefore, the factor $\frac{\text{C}\text{x, N=0}}{\text{Y}\text{inoc, ph}}$ was included in Equation S20 assuming that for the batch the inoculum, which is defined as the biomass present at the onset of nitrogen starvation (*C_x, N= 0_*), was produced at a certain biomass yield on light (*Y_inoc, ph_*) in a continuous-operated PBR at a biomass concentration equal to *C_x, N= 0_*. Differently, for (long-term) repeated-batch cultivations, it can be assumed that inoculum production would be required only at the start of the cultivation and thus it can be neglected. Hence, $\frac{\text{C}\text{x, N=0}}{\text{Y}\text{inoc, ph}}$ was set to zero for repeated-batch.

$\text{TAG yield on light = }\frac{\text{TAG (t)}}{\frac{\text{I}\text{0}\text{ t}}{\text{z}}\text{+ }\frac{\text{C}\text{x, N=0}}{\text{Y}\text{inoc, ph}}}$ Eq. S20

Differently from the original model of [1], in which the inoculum of the batch cultivation was always produced at a *Y_inoc, ph_* of 1 g mol_ph_^-1^, in our modified model, *Y_inoc, ph_* was calculated as a function of incident light intensity, biomass concentration and reactor light path (Eq. S21), as the dynamics of cell recovery and thus the re-growth phase in repeated-batch cycles are also dependent on these parameters.

$\text{Y}\text{inoc}\text{, ph }\text{= }\frac{\text{Y}\text{x, ph}\text{ ∙}\bar{\text{q}\text{ph}}\text{ - m}\text{s }}{\frac{\text{I}\text{0}}{\text{z ∙ C}\text{x, N=0}}}$ Eq. S21

**1.2 Model calibration for nitrogen run-out batch cultivations of *Nannochloropsis* sp.**

Upon the adaptations made as described in section 1.1, the model of [1] was calibrated using the results of our nitrogen (N) run-out batch experiment. The results of the duplicate cultivations were combined and treated, by averaging, as a single dataset. The first experimental point, immediately after setting the light at 636 µmol m^-2^ s^-1^, was used as initial condition for integration and was considered as t = 0. The following data points, until biomass decay was observed, were used for parameter estimation (MATLAB: Monte-Carlo algorithm and *fminsearch*) as described by [1], yielding the calibrated values as presented in Table S1. The experimental settings for incident light intensity and reactor light path, and the experimentally observed absorption cross-section and cellular nitrogen content of N-replete cells were used as model constants.

**Table** S**1 Nitrogen-run-out batch.**

Estimated parameters, constants and boundary conditions used for solving the material balances presented by [1] for batch TAG production.

| **Estimated parameters** | **Unit** | **Value** |
| --- | --- | --- |
| q_ph_^max, replete^ | mol g^-1^ h^-1^ | 0.026 |
| Q_min_ | g g^-1^ | 0.020 |
| m_s_ | mmol g^-1^ h^-1^ | 0.093 |
| X_cho_ | g g^-1^ | 0.310 |
| **Constants** | **Unit** | **Value** |
| Q_max_ | g g^-1^ | 0.070 |
| a_replete_ | m^2^ g^-1^ | 0.080 |
| z | m | 0.020 |
| I_0_ | µmol m^-2^ s^-1^ | 636 |
| X_CHO, x_ | g g^-1^ | 0.180 |
| X_TAG, x_ | g g^-1^ | 0.080 |
| **Initial values** | **Unit** | **Value** |
| C_x_ | g m^-3^ | 928 |
| TAG and CHO | g m^-3^ | 0 |
| N-NO_3_ | g m^-3^ | 107 |

**1.3 Model validation for repeated-batch cultivations**

The model was validated on the repeated-batch cultivations using the calibrated values of the batch experiments, with the exception of *X_cho_*. The two repeated-batch cultivations led to similar residual biomass fractions (*X_cho_*). However, these were much lower compared to *X_cho_* during the batch (Table S1). Consequently, the parameter estimation procedure was performed on *X_cho_* only, while the maximum photosynthetic rate of nitrogen-replete cells (*q_ph_^max, replete^*), the minimum cellular nitrogen content (*Q_min_*) and the maintenance coefficient (*m_s_*) were fixed to the estimated values from the batch model. Regardless of the repeated-batch dataset used, an estimated value of 0.17 g g^-1^ was obtained for *X_cho_*.

A TAG degradation mechanism was devised (Fig. 7) and then implemented in the model (Equations S 7 -8 and S10). Although net TAG degradation was not observed with the tested repeated-batch cultivations (Fig. 3C and Fig. 4C), this did occur in the N-rich medium-replenished batch cultivation (Fig. 2C). Thus, the values for the critical cellular nitrogen content at which TAG degradation commences (*Q_deg_*) and the TAG degradation rate (*r_TAG, x_*) were estimated from the N-rich medium-replenished batch cultivation.

For each of the two repeated-batch cultivations, the first experimental point, immediately after setting the light at 636 µmol m^-2^ s^-1^, was used as initial condition for integration and was considered as t = 0.

**Table** S**2 Repeated-batch model.**

Estimated parameter, constants and boundary conditions used for solving the material balances for repeated-batch TAG production.

| **Estimated parameter** | **Unit** | **Value** |
| --- | --- | --- |
| X_cho_ | g g^-1^ | 0.170 |
| **Constants** | **Unit** | **Value** |
| q_ph_^max, replete^ | mol g^-1^ h^-1^ | 0.026 |
| Q_min_ | g g^-1^ | 0.020 |
| m_s_ | mmol g^-1^ h^-1^ | 0.093 |
| Q_max_ | g g^-1^ | 0.070 |
| a_replete_ | m^2^ g^-1^ | 0.080 |
| z | m | 0.020 |
| I_0_ | µmol m^-2^ s^-1^ | 636 |
| X_CHO, x_ | g g^-1^ | 0.180 |
| X_TAG, x_ | g g^-1^ | 0.080 |
| r_TAG, x_ | g g^-1^ h^-1^ | 0.011 |
| Q_deg_ | g g^-1^ | 0.025 |
| **Initial values** | **Unit** | **Value** |
| C_x_ | g m^-3^ | 1173 (LN); 900 (HN) |
| TAG and CHO | g m^-3^ | 0 |
| N-NO_3_ | g m^-3^ | 65 (LN); 100 (HN) |

**1.4 List of symbols**

| **Symbol** | **Unit** | **Description** |
| --- | --- | --- |
| a | m^2^ g^-1^ | Absorption cross-section |
| a_replete_ | m^2^ g^-1^ | Absorption cross-section of nitrogen replete biomass |
| CHO | g m^-3^ | Concentration of residual biomass made during N-starvation |
| C_x_ | g m^-3^ | Biomass concentration |
| C_x, N = 0_ | g m^-3^ | Biomass concentration at onset of nitrogen starvation |
| f | % (v/v) | Remaining culture fraction in the reactor after harvest |
| I_0_ | mol m^-2^ s^-1^ | Incident light intensity |
| m_s_ | mmol g^-1^ h^-1^ | Maintenance coefficient |
| N | g m^-3^ | Amount of nitrogen resupplied after each harvest |
| N-NO_3_^-^ | g m^-3^ | Extracellular nitrate concentration |
| *Q* | g g^-1^ | Cellular nitrogen content |
| Q_deg_ | g g^-1^ | Cellular nitrogen content below which TAG degradation begins |
| Q_max_ | g g^-1^ | Maximum cellular nitrogen content of N-replete biomass |
| Q_min_ | g g^-1^ | Minimum cellular nitrogen content |
| $\bar{\text{q}\text{ph}}$ | mol g^-1^ h^-1^ | Biomass-specific photosynthetic rate averaged throughout the reactor |
| q_ph_^max, replete^ | mol g^-1^ h^-1^ | Maximum photosynthetic rate of N-replete cells |
| q_i_ | g g^-1^ h^-1^ | Biomass specific production rate of component i |
| r_TAG, x_ | g g^-1^ h^-1^ | Conversion rate of TAG into reproducing biomass |
| t | h | Time |
| TAG | g m^-3^ | TAG concentration |
| X_cho, x_ | g g^-1^ | CHO content in reproducing biomass |
| X_cho_ | g g^-1^ | CHO content in newly formed biomass during N-starvation, excluding reproducing biomass |
| X_TAG, x_ | g g^-1^ | TAG content in reproducing biomass |
| *Y_i, j_* | g mol_ph_^-1^ or g g^-1^ | Yield of component *i* on component *j* (subscript *ph* refers to photon) |
| z | m | Reactor light path |
| Δ | h | Repeated-batch cycle duration |

**1.5 References**

1. Breuer G, Lamers PP, Janssen M, Wijffels RH, Martens DE: **Opportunities to improve the areal oil productivity of microalgae**. *Bioresour Technol* 2015, **186**:294–302.

2. Jassby AD, Platt T: **Mathematical formulation of the relationship between photosynthesis and light for phytoplankton**. *Limnol Oceanogr* 1976, **21**:540–547.

3. Geider RJ, MacIntyre HL, Kana TM: **A dynamic regulatory model of phytoplanktonic acclimation to light, nutrients, and temperature**. *Limnol Oceanogr* 1998, **43**:679–694.

4. Benvenuti G, Bosma R, Cuaresma M: **Selecting microalgae with high lipid productivity and photosynthetic activity under nitrogen starvation**. *J Appl Phycol* 2014, **27**:1425 – 1431.

5. Droop MR: **Vitamin B12 and Marine Ecology. IV. The Kinetics of Uptake, Growth and Inhibition in Monochrysis Lutheri**. *J Mar Biol Assoc United Kingdom* 2009, **48**:689.

6. Sukenik A, Bennett J, Falkowski P: **Light-saturated photosynthesis — Limitation by electron transport or carbon fixation?** *Biochim Biophys Acta - Bioenerg* 1987, **891**:205–215.

7. Kliphuis AMJ, Klok AJ, Martens DE, Lamers PP, Janssen M, Wijffels RH: **Metabolic modeling of Chlamydomonas reinhardtii: energy requirements for photoautotrophic growth and maintenance.** *J Appl Phycol* 2012, **24**:253–266.
