# Supplementary material for: Microalgal TAG production strategies: why batch beats repeated-batch
Source: Biotechnol Biofuels. 2016 Mar 16;9:64. doi: 10.1186/s13068-016-0475-4 (PMC4793540; doi:10.1186/s13068-016-0475-4)
Supplement: Supplementary file 2 — 10.1186/s13068-016-0475-4 Nitrogen uptake, biomass and TAG production in repeated-batch cycles. The time at which nitrogen was depleted from the medium, the biomass-specific nitrogen uptake and TAG production rates, as well as the time-averaged biomass and TAG yields on light at the harvest of each cycle are shown for the two repeated-batch cultivations. [file 13068_2016_475_MOESM2_ESM.docx]

**Additional file 2 Nitrogen uptake, biomass and TAG production in repeated-batch cycles**

Time of nitrogen-depletion (*t_N = 0_*), biomass-specific nitrogen uptake (*-q_N_*) and TAG production (*q_TAG_*) rates, time-averaged biomass (*Y_x, ph, HARV, CYCLE_ (t)*) and TAG (*Y_TAG, ph, HARV, CYCLE_ (t)*) yields on light at the harvest of each cycle for the 70 and 140 mg L^-1^ nitrogen resupply repeated-batch cultivations are shown. Biomass specific rates are calculated by normalizing the volumetric rates to the average biomass concentration during the considered time interval. Volumetric rates are calculated with linear regression of concentrations vs. time. R^2^ of linear regressions are always > 0.90. Productivities are corrected for the amount of biomass and TAGs present at the start of each cycle (i.e. t = 0). Constant cycle repetitions are highlighted in bold. The standard deviation is for all cases within 5% of average.

| *70 mg N-NO_3_^-^ L^-1^ repeated-batch cultivation (70N)* | | | | | |
| --- | --- | --- | --- | --- | --- |
|  | *t_N = 0_*  (h) | *-q_N_*  (mg g^-1^ h^-1^) | *q_TAG_*  (mg g^-1^ h^-1^) | *Y_x, ph, HARV, CYCLE_ (t)*  (g mol_ph_^-1^) | *Y_TAG, ph, HARV, CYCLE_ (t)*  (g mol_ph_^-1^) |
| cycle #0 | 13 | 2.7 | 9.6 | 0.29 | 0.14 |
| cycle #1 | 18 | 1.4 | 7.9 ^*^ | 0.25 | 0.12 |
| **cycle #2** | **18** | **1.3** | **6.5 ^*^** | **0.25** | **0.13** |
| **cycle #3** | **17** | **1.5** | **6.5 ^*^** | **0.25** | **0.13** |
| **cycle #4** | **17** | **1.4** | **6.8 ^*^** | **0.25** | **0.13** |
| *140 mg N-NO_3_^-^ L^-1^ repeated-batch cultivation (140N)* | | | | | |
|  | *t_N = 0_*  (h) | *-q_N_*  (mg g^-1^ h^-1^) | *q_TAG_*  (mg g^-1^ h^-1^) | *Y_x, ph, HARV, CYCLE_ (t)*  (g mol_ph_^-1^) | *Y_TAG, ph, HARV, CYCLE_ (t)*  (g mol_ph_^-1^) |
| cycle #0 | 14 | 3.0 | 5.9 | 0.32 | 0.10 |
| cycle #1 | 14 | 2.5 | 5.3 **^**^** | 0.28 | 0.10 |
| cycle #2 | 16 | 2.3 | 4.5 **^**^** | 0.31 | 0.11 |
| cycle #3 | 16 | 2.0 | 4.5 **^**^** | 0.28 | 0.10 |
| cycle #4 | 18 | 1.7 | 4.8 **^**^** | 0.27 | 0.11 |
| cycle #5 | 19 | 2.0 | 7.2 **^**^** | 0.28 | 0.12 |
| **cycle #6** | **22** | **1.7** | **6.8 ^**^** | **0.30** | **0.12** |
| **cycle #7** | **21** | **1.7** | **6.8 ^**^** | **0.30** | **0.12** |
| **cycle #8** | **21** | **1.8** | **6.6 ^**^** | **0.30** | **0.12** |

(^*^) Calculated excluding the first 24 hours of the cycle

(^**^) Calculated excluding the first 30 hours of the cycle
