# Supplementary material for: Microalgal TAG production strategies: why batch beats repeated-batch
Source: Biotechnol Biofuels. 2016 Mar 16;9:64. doi: 10.1186/s13068-016-0475-4 (PMC4793540; doi:10.1186/s13068-016-0475-4)
Supplement: Supplementary file 3 — 10.1186/s13068-016-0475-4 Maximum photosystem II efficiency in batch and repeated-batch. Time-evolution of maximum photosystem II efficiency is shown for the batch and the repeated-batch cultivations. [file 13068_2016_475_MOESM3_ESM.docx]

**Additional file 3 Maximum photosystem II efficiency in batch and repeated-batch cultivations**

Time-evolution of maximum photosystem II efficiency (*F_v_/F_m_*) is shown for the batch and the 70 (70N) and 140 (140N) mg N-NO_3_^-^ L^-1^ repeated-batch cultivations. Red symbols indicate the value of maximum photosystem II efficiency at N-rich medium resupply.
