# Supplementary material for: Microalgal TAG production strategies: why batch beats repeated-batch
Source: Biotechnol Biofuels. 2016 Mar 16;9:64. doi: 10.1186/s13068-016-0475-4 (PMC4793540; doi:10.1186/s13068-016-0475-4)
Supplement: Supplementary file 4 — 10.1186/s13068-016-0475-4 Effect of biomass concentration and reactor light path on batch TAG yield on light. The effect of biomass concentration at the onset of nitrogen starvation and reactor light path on the maximum TAG yield on light of batch processes is shown. [file 13068_2016_475_MOESM4_ESM.docx]

**Additional file 4** **Effect of biomass concentration and reactor light path on batch TAG yield on light**

The output of Monte-Carlo-sampled simulations on maximum TAG yield on light (blue symbols is shown. The ranges within which the parameters were varied are given in Table 3. **A**) Impact of incident light intensity, biomass concentration at onset of nitrogen starvation (*C_x, N = 0_*) and reactor light path (*z*). **(B- C**) To illustrate the contribution of *C_x, N = 0_* and *z* independently from the influence of light, the TAG yield was normalized to the yield predicted at the same incident light intensity and with the value of the parameter under study as presented in Table S1 (red symbols).
