# Supplementary material for: Microalgal TAG production strategies: why batch beats repeated-batch
Source: Biotechnol Biofuels. 2016 Mar 16;9:64. doi: 10.1186/s13068-016-0475-4 (PMC4793540; doi:10.1186/s13068-016-0475-4)
Supplement: Supplementary file 5 — 10.1186/s13068-016-0475-4 Impact of maintenance coefficient on accuracy of model predictions. The impact of the estimated maintenance coefficient on the predicted TAG yield on light is investigated and discussed. [file 13068_2016_475_MOESM5_ESM.docx]

**Additional file 5 Impact of maintenance coefficient on accuracy of model predictions**

The estimated maintenance coefficient (m_s_) of this study (0.093 mmol_ph_ g^-1^ h^-1^, Additional file 1: Sect. S1.2) is lower than the m_s_ reported for *Chlamydomonas reinhardtii* (3.08 mmol_ph_ g^-1^ h^-1^) [1] , *Chlorella sorokiniana* (2.84 mmol_ph_ g^-1^ h^-1^) [2] and *Nannochloropsis salina* (7.62 mmol_ph_ g^-1^ h^-1^) [3] under N-replete conditions. Two possible explanations for such difference are: 1) The published maintenance coefficient include the efficiency of photosynthesis during the measurements and will thus, due to inherent losses, have a higher requirement compared to our maintenance coefficient, which is modeled separately from photosynthesis. 2) Likely the maintenance coefficient of N-starved cells is lower than that of N-replete cells, possibly because of a reduced metabolic activity.

Nevertheless, the model is able to adequately describe the experimental data (Fig. S5.1) when the average of the published maintenance coefficients (i.e. 4.51 mmol g^-1^ h^-1^) was used and the other model parameters were fitted on our batch nitrogen run-out dataset. Parameter estimation was performed as described in Additional file 1: Sect. S1.2, yielding values of 0.038 mol g^-1^ h^-1^ for the maximum photosynthetic rate of nitrogen-replete cells, 0.016 g g^-1^ for the minimum cellular nitrogen content and 0.31 (batch) or 0.17 (repeated-batch) g g^-1^ for the residual biomass fraction made during nitrogen starvation.

Furthermore, we found that, for the original set of model parameters, the maintenance coefficient would have to be increased by a factor 12 to 25 in order to have a 10 - 15% reduction in TAG yield on light (Fig. S5.2). This shows that the model is not very sensitive to the maintenance coefficient.


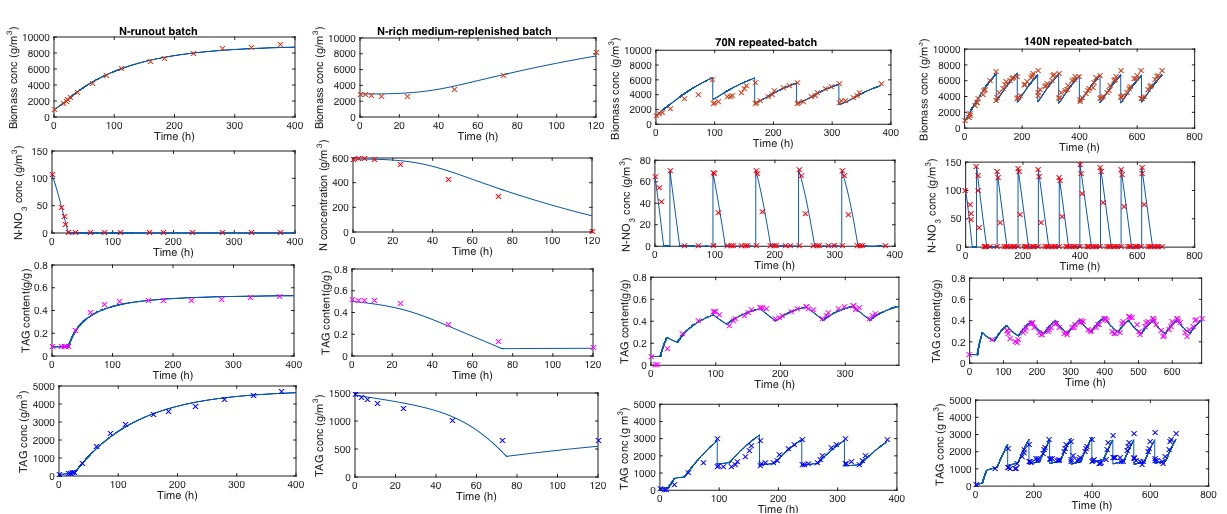


**Figure S5.1** **Model simulations and experimental data**

Model simulations (lines) and experimental data (symbols) of biomass, external N-NO_3_^-^ and TAG concentrations as well as TAG content for the N-run-out batch, N-rich medium-replenished batch, 70 mg_N_ L^-1^-rich medium (70N) repeated-batch and 140 mg_N_ L^-1^-rich medium (140N) repeated-batch cultivations. As described in the main text, model simulations were performed fixing the maintenance coefficient to the average value reported in literature.

**Figure S5.2 Output of Monte-Carlo-sampled simulations**

**(A)** Impact of light intensity and maintenance coefficient (*m_s_*) on batch TAG yield on light. Red symbols represent the TAG yield on light calculated keeping *m_s_* at the value estimated in our study To illustrate the individual contribution of *m_s_* **(B)**, the TAG yield is normalized to the yield (red symbols) predicted at the same incident light intensity and with the value of *m_s_* as estimated in our study.

**References**

1. Vejrazka C, Janssen M, Benvenuti G, Streefland M, Wijffels RH: **Photosynthetic efficiency and oxygen evolution of Chlamydomonas reinhardtii under continuous and flashing light**. *Appl Microbiol Biotechnol* 2013, **97**:1523–1532.

2. Kliphuis AMJ, Janssen M, van den End EJ, Martens DE, Wijffels RH: **Light respiration in Chlorella sorokiniana.** *J Appl Phycol* 2011, **23**:935–947.

3. Sforza E, Calvaruso C, Meneghesso A, Morosinotto T, Bertucco A: **Effect of specific light supply rate on photosynthetic efficiency of Nannochloropsis salina in a continuous flat plate photobioreactor.** *Appl Microbiol Biotechnol* 2015, **99**:8309–18.
