# Supplementary material for: Microalgal TAG production strategies: why batch beats repeated-batch
Source: Biotechnol Biofuels. 2016 Mar 16;9:64. doi: 10.1186/s13068-016-0475-4 (PMC4793540; doi:10.1186/s13068-016-0475-4)
Supplement: Supplementary file 6 — 10.1186/s13068-016-0475-4 Optimized model scenarios and corresponding parameters. Optimized TAG yields on light and TAG contents and corresponding model parameter values at which these were achieved are shown for batch and repeated-batch processes. [file 13068_2016_475_MOESM6_ESM.docx]

**Additional file 6 Optimized model scenarios and corresponding parameters**

Optimized TAG yields on light and TAG contents and corresponding model parameter values at which these were achieved. **B**: batch; **RB**: repeated-batch. High (**HL**), intermediate (**IL**) and low (**LL**) light intensities correspond to incident light intensities of 1500, 600 and 200 μmol m^-2^ s^-1^, respectively.

| **Scenario** | | **TAG yield**  **on light**  (g mol_ph_^-1^) | **TAG content**  (g g^-1^) | ***z***  (m) | ***C_x, N = 0_***  (g m^-3^) | | ***X_cho_***  (g g^-1^) | ***q_ph_^max, replete^***  (mol g^-1^ h^-1^) |
| --- | --- | --- | --- | --- | --- | --- | --- | --- |
| 1B | HL (Base case) | 0.12 | 0.42 | 0.02 | 2451 | | 0.31 | 0.026 |
| 2B | IL | 0.23 | 0.42 |  |  |  |  |  |
| 3B | LL | 0.41 | 0.43 |  |  |  |  |  |
| 4B | Increased maximum photosynthetic rate and decreased residual biomass fraction  (HL) | 0.18 | 0.52 |  |  |  | 0.12 | 0.037 |
| 5B | Increased maximum photosynthetic rate and decreased residual biomass fraction  (IL) | 0.31 | 0.52 |  |  |  | 0.13 | 0.034 |
| 6B | Increased maximum photosynthetic rate and decreased residual biomass fraction  (LL) | 0.49 | 0.53 |  |  |  | 0.11 | 0.030 |
| **Scenario** | | **TAG yield**  **on light**  (g mol_ph_^-1^) | **TAG content**  (g g^-1^) | ***N***  (g m^-3^) | ***f***  (%) | ***Δ***  (hours) | ***X_cho_***  (g g^-1^) | ***q_ph_^max, replete^***  (mol g^-1^ h^-1^) |
| 1RB | HL (Base case) | 0.07 | 0.54 | 70 | 0.50 | 72 | 0.17 | 0.026 |
| 2RB | IL | 0.15 | 0.52 |  |  |  |  |  |
| 3RB | LL | 0.29 | 0.44 |  |  |  |  |  |
| 4RB | Optimal N-resupply, cycle duration and harvest volume  (HL) | 0.09 | 0.50 | 134 | 0.29 | 85 | 0.17 | 0.026 |
| 5RB | Optimal N-resupply, cycle duration and harvest volume  (IL) | 0.18 | 0.51 | 171 | 0.46 | 134 |  |  |
| 6RB | Optimal N-resupply, cycle duration and harvest volume  (LL) | 0.33 | 0.48 | 142 | 0.38 | 155 |  |  |
| 7RB | Increased maximum photosynthetic rate and decreased residual biomass fraction  (HL) | 0.10 | 0.60 | 70 | 0.50 | 72 | 0.11 | 0.038 |
| 8RB | Increased maximum photosynthetic rate and decreased residual biomass fraction  (IL) | 0.19 | 0.54 |  |  |  | 0.17 | 0.037 |
| 9RB | Increased maximum photosynthetic rate and decreased residual biomass fraction  (LL) | 0.34 | 0.49 |  |  |  | 0.13 | 0.038 |
| 10RB | Optimal N-resupply, cycle duration and harvest volume & Increased maximum photosynthetic rate and decreased residual biomass fraction  (HL) | 0.11 | 0.35 | 167 | 0.21 | 47 | 0.25 | 0.038 |
| 11RB | Optimal N-resupply, cycle duration and harvest volume & Increased maximum photosynthetic rate and decreased residual biomass fraction  (IL) | 0.22 | 0.47 | 161 | 0.14 | 87 | 0.15 | 0.037 |
| 12RB | Optimal N-resupply, cycle duration and harvest volume & Increased maximum photosynthetic rate and decreased residual biomass fraction  (LL) | 0.39 | 0.48 | 177 | 0.29 | 133 | 0.13 | 0.038 |
